# Crystal structure and polymorphic forms of auranofin revisited†

**DOI:** 10.1039/d5ra00196j

**Published:** 2025-04-03

**Authors:** Marcin Ziemniak, Damian Trzybiński, Sylwia Pawlędzio, Gabriela Filipowicz, Beata Pająk-Tarnacka, Waldemar Priebe, Krzysztof Woźniak

**Affiliations:** a Biological and Chemical Research Centre, Department of Chemistry, University of Warsaw Żwirki i Wigury 101 Warsaw 02-089 Poland mziemniak@chem.uw.edu.pl dtrzybinski@cnbc.uw.edu.pl kwozniak@chem.uw.edu.pl; b Neutron Scattering Division, Oak Ridge National Laboratory Oak Ridge Tennessee 37831 USA pawledzios@ornl.gov; c Pritzker School of Molecular Engineering, The University of Chicago Chicago Illinois 60637 USA gfilippo38@gmail.com; d WPD Pharmaceuticals Inc Żwirki I Wigury 101 Warsaw 02-089 Poland bepaj@wp.pl; e Department of Experimental Therapeutics, The University of Texas MD Anderson Cancer Center 1901 East Rd. Houston TX 77054 USA wpriebe@mac.com

## Abstract

Auranofin, initially developed as a treatment for rheumatoid arthritis, is currently under extensive investigation as a potential drug for various conditions, including cancer, bacterial infections, and parasitic infections. The compound is a known inhibitor of thioredoxin reductase (TXNRD1) and related selenoproteins. Although preliminary studies on the auranofin crystal polymorphism exist, and a low-quality crystal structure has been reported, a comprehensive crystallographic characterization remains unexplored. Utilizing X-ray crystallography techniques, we conducted detailed structural analysis of auranofin and compared our findings with related organogold compounds. Implementation of Hirshfeld atom refinement (HAR) enabled a more accurate hydrogen atom positioning in the structure. The crystal packing reveals a layered arrangement stabilised by numerous weak hydrogen bonds and dispersive interactions. Notably, our attempts to reproduce the previously reported polymorphic form of auranofin, purportedly more water-soluble, were unsuccessful despite following published protocols. To our knowledge, this is the first study describing a “disappearing polymorph” phenomenon of any pharmaceutically relevant transition metal coordination compound. Our findings may have significant implications for medicinal chemistry and pharmacology of coordination complexes, suggesting the need for systematic revision of historical crystallographic data in this field.

## Abstract 2

Structural studies of auranofin showing the first case of a “disappearing polymorph” phenomenon for a pharmaceutically relevant transition metal coordination compound.

## Graphical abstract

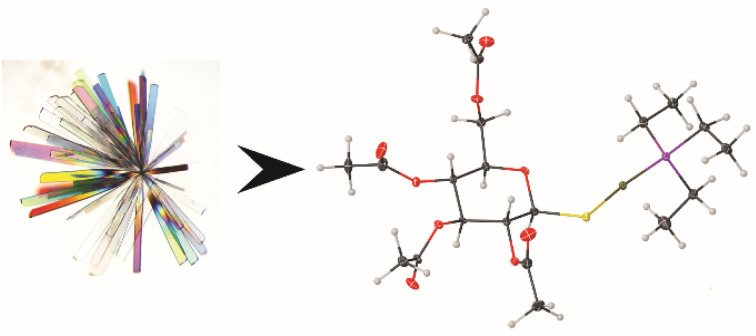

## 1. Introduction

**Scheme 1:**
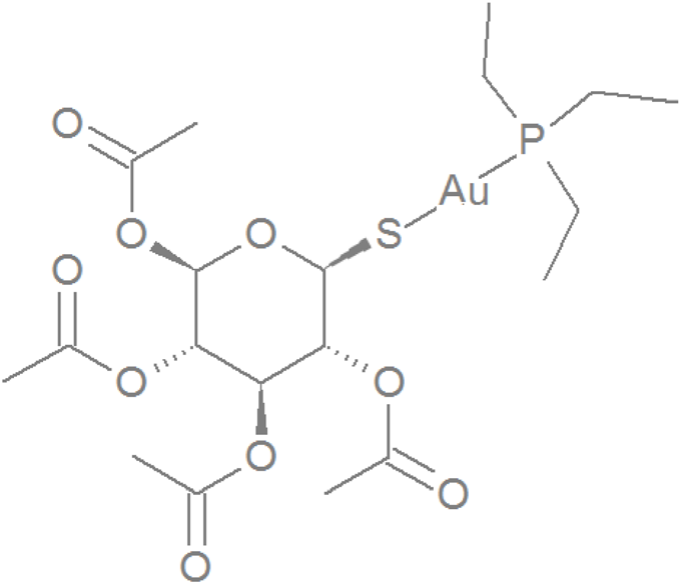
The structural formula of auranofin (β anomer).

Gold compounds have been used in medicine since the beginning of the 20th century. One of the more widespread drugs of this type is auranofin (AF), which was developed in the 1970s as a remedy for rheumatoid arthritis (Scheme 1).^1,2^ Despite its potency, AF has largely been superseded in clinical practice by other antirheumatic drugs, primarily gold sodium thiomalate and related compounds, as well as methotrexate and sulfasalazine.^1^ The major reason for this shift was the fear of potential long-term side effects due to immune suppression. Although its use in clinical practice has decreased, recent findings indicated the applicability of AF for treating various diseases, including cancer and bacterial and parasitic infections in humans, livestock, and pets.^3–7^ Numerous studies published in recent years show that this compound is able to induce cell death in various types of cancer, including very aggressive forms, such as TNBC (triple-negative breast cancer),^8^ adenocarcinoma,^9^ osteosarcoma,^10^ lung cancer,^11^ and brain tumours,^12^ among others. Another potential clinical application of AF is treatment of bacterial infections caused by drug-resistant strains of bacteria including *M. tuberculosis*, *B. subtilis*, *E. faecalis*, and *Enterococcus casseliflavus* as well as drug-resistant strains of *E. faecium*, *S. aureus*, *Streptococcus pyogenes*, and *Clostridium difficile*.^13^ Furthermore, it has been demonstrated that AF can be potentially repurposed to treat parasitic diseases including giardiasis,^14^ filariasis, onchocerciasis^15^ and fungal infections.^16,17^ Notably, some of these diseases are neglected tropical, or orphan diseases (*i.e.*, osteosarcoma, mentioned earlier). Financial restraints often limit research of new drugs for these conditions.

A primary mechanism of AF action is the inhibition of thioredoxin reductases activity (TXNRD), which is represented by two isoforms: a cytoplasmic TrxR1 form and a mitochondrial TrxR2 form. These enzymes play a major role in regulating reactive oxygen species (ROS) levels, thus protecting cells from the harmful effects of oxidative stress. Inhibition of TXNRD increases cellular oxidative stress and induces apoptosis, especially in cancer cells.^18^ TXNRD overexpression is often associated with aggressive tumour progression and poor patient survival rates. As such, the thioredoxin system itself is a promising target for new anticancer therapies.^4,19^ Interestingly, a recent chemical proteomics study confirmed TXNRD1 as the main AF target, with the primary mechanism of action being the perturbation of oxidoreductase pathways.^20^ Apart from the thioredoxin system, other molecular targets of AF were reported, including deubiquitinases (DUBs),^21^ predominantly proteasomal proteins USP14 and UCHL5,^22^ and hexokinase.^23^ Studies in both cell lines and murine models have demonstrated that high doses of AF induce ferroptosis – a form of regulated cell death caused by iron-dependent oxidative disruption of cellular membranes – and promote lipid peroxidation through TXNRD1 inhibition. Moreover, AF inhibits several cancer signalling pathways, contributing to its antiproliferative effects. For example, AF blocks signal transducer and activator of transcription 3 (STAT3)-dependent NF-kB and telomerase activity in breast cancer and multiple myeloma cells.^24,25^AF also activates the FOXO3 suppressor and inhibits the protein kinase Cι (PKCι) signalling in ovarian cancer models, as well as the PI3K/AKT/mTOR axis in non-small cell lung cancer cells.^26,27^

These findings prompted the development of novel Au(i) complexes incorporating pyranose subunits, which demonstrated promising anticancer activity.^28^ While initial efforts focused on phosphine complexes structurally analogous to AF, subsequent research explored N-heterocyclic carbene ligands, which combine synthetic accessibility with the ability to fine-tune their biological properties.^29,30^ Despite structural differences among Au(i) complexes, in each case the biologically active fragment is the gold(i) cation, capable of forming covalent bonds with exposed cysteine or selenocysteine residues of the target protein. The compound itself is a carrier responsible for delivering the gold ion to its destination. Therefore, the solubility and physiological stability of the carrier, as well as potential off-target interactions, have a decisive impact on their medical properties. Both AF and related organogold compounds act as irreversible inhibitors of thioredoxin reductase and other selenoenzymes. However, the precise molecular mechanism of inhibition remains incompletely understood and requires further biochemical investigation.^31,32^

The structural chemistry of glucose-derived gold complexes and related compounds is relatively well-documented, however, with several crystal structures deposited in the Cambridge Structural Database (CSD). The first crystal structure of AF (CCDC† entry: AGLPAU),^33^ reported approximately forty years ago, was of low quality and lacked defined hydrogen atom positions. A comprehensive characterization of its crystal structure and intermolecular interactions has not been available until this study. Previous literature reports suggest the existence of an alternative AF polymorph with enhanced aqueous solubility, a property of significant pharmacological interest.^34,35^ This polymorph is expected to crystallise from a mixture of cyclohexane and ethyl acetate, whereas commonly known AF crystals (denoted here as the A polymorph) are usually obtained by crystallisation from low molecular weight alcohols. However, only limited NMR and calorimetric data support the existence of the second polymorph, and no additional studies were aimed at resolving this issue.^35^

This observation may represent a case of “disappearing polymorphs” – a documented phenomenon where a previously reported crystalline form becomes impossible to reproduce under identical crystallization conditions.^36,37^ Although this phenomenon has been extensively investigated across various chemical systems, the mechanistic basis for polymorph disappearance remains to be fully elucidated. It is hypothesised that minute changes the nucleation and crystal growth processes caused by trace impurities, subtle variations in environmental conditions, and differences in crystallization methodology are major contributing factors.^36,38^ Disappearing polymorphs presents significant challenges in pharmaceutical manufacturing, as it can affect drug properties and necessitate new methodologies to reproducibly obtain clinically relevant yet metastable forms. Notable instances include ritonavir,^39^ an antiretroviral drug used in HIV treatment, and salts of paroxetine, a common antidepressant.^36,40^

The primary objectives of this study were to obtain a good-quality crystal structure of AF, investigate the intermolecular interactions within its crystal, and seek further evidence for the existence of its other polymorphs. Despite numerous attempts, we were unable to identify any new polymorphic structures, which suggests that previous reports may fall into the category of “disappearing polymorph” phenomenon. Thus, it seems rather unlikely that the second crystal form could be prepared again. Nonetheless, we acquired a high-quality X-ray crystal structure of the canonical polymorph of AF, which was then refined using Hirshfeld atom refinement (HAR). We characterised its supramolecular landscape with particular attention on the H-bond network, and we also performed computational studies on crystals of AF and some related compounds to obtain more information on the energetic characteristics and intermolecular interactions within these structures. This analysis included cohesive energies of the crystal lattice, Hirshfeld surface (HS) analysis, and qualitative charge-density studies. Additionally, we compared the chemical environment of gold cations in crystals of pharmaceutically relevant compounds and selected proteins.

## 2. Results and discussion

### 2.1. Crystallization and crystal morphology

**Fig. 1:**
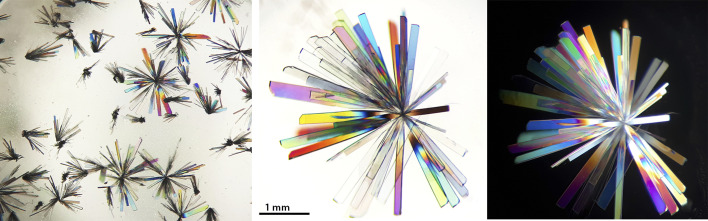
Exemplary crystals of canonical AF polymorph illuminated in a polarised optical microscope.

AF was crystallised from ethanol under ambient conditions. Colourless crystals appeared after a few days, and most were of high quality providing discernible diffraction patterns. Nonetheless, their diffraction limit was 0.7 Å, making them unsuitable for high-resolution measurements for charge density studies. All observed crystals display an acicular or columnar habit and form radial clusters (Fig. 1). Unfortunately, we were unable to obtain any crystals of the second AF polymorph (dubbed “auranofin B” in the literature) using methods described in previous publications, *i.e.*, crystallization from a mixture of cyclohexane and ethyl acetate. Systematic modifications of crystallization conditions, including various non-polar solvent combinations, temperature control (4–25 °C), and different container materials (glass and plastic of various volumes and shapes), exclusively produced the canonical A polymorph. Experiments performed in different rooms, where AF has never been stored, also did not result in polymorph B crystals. We performed an X-ray diffraction screening testing crystals of various sizes and shapes, but all were found to be the A polymorph. Considering the possibility that polymorph B might exist as microcrystals or as inclusions within polymorph A, we conducted systematic analyses of bulk AF samples recrystallized from different solvent systems. In each case crystallographic characterization consistently confirmed the exclusive presence of polymorph A.

In our opinion, this is a typical case of “disappearing polymorphs” – crystal forms that have been previously observed experimentally but later became difficult or even impossible to obtain again. In most cases, such a situation is caused by the formation of a late appearing, more stable crystal form, which subsequently eradicates the first polymorph. The phenomenon of disappearing polymorphs has been primarily documented in organic systems, with only a single reported case involving a coordination compound.^41^ This previous study described a mechanochemically synthesised Hg-containing metal–organic framework that produced a thermodynamically preferred topological polymorph. To our knowledge, the current study represents the first documented instance of a disappearing polymorph among medicinally relevant coordination compounds. The case of auranofin is also important since polymorph B is believed to have better solubility in water and may have better bioavailability than the canonical form. Moreover, these results suggest that further research and re-examining older studies on the polymorphism of drugs may lead to significant findings.

### 2.2. X-ray diffraction studies

**Fig. 2:**
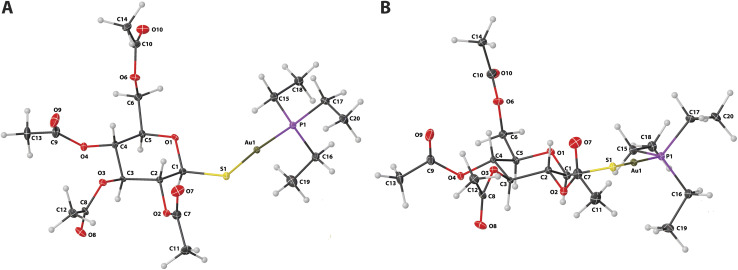
The content of the asymmetric unit of the crystal lattice of AF with the atom numbering scheme from top (A) and (B) lateral projections. Atomic displacement ellipsoids are shown with a 25% probability level, and the hydrogen atoms are shown as small spheres of an arbitrary radius.

Single-crystal X-ray diffraction analysis confirmed the postulated structure of the AF, which crystallises in the monoclinic *P*2_1_ space group, and the asymmetric unit of its crystal lattice is composed of one molecule (Fig. 2). All refinement parameters and other geometrical parameters are summarised in ESI (Tables S1–S8†). Spherical refinement based on Independent Atom Model (IAM) was conducted in *ShelXL* program,^42^ while the non-spherical atom refinement was carried out using *NoSpherA2* (PBE/x2c-TVP level of theory)^43–45^ implemented in *Olex2* software.^46^ The molecular wavefunction was computed for an isolated molecule (HAR-single), or a AF trimer (HAR-cluster). In both strategies a relativistic correction was taken into account by applying the DKH2 Hamiltonian.^47^ Despite methodological differences between these approaches (IAM and various flavours of HAR), only a moderate improvement of the overall model quality was observed (Table S9†). Although HAR provided better positioning of hydrogen atoms, which allowed to reduce the number of constraints imposed on the model even if the final resolution was modest (0.84 Å), the results are virtually identical no matter which variant of HAR was used. Moreover, to achieve optimal geometry of methyl groups it was necessary to impose distances for hydrogen atoms based on neutron diffraction experiments. The application of higher-grade hybrid or range-separated functionals, such as M062X^48^ or ωB97X,^49^ also did not improve the model quality and was more computationally demanding (data not shown). To summarise, in our opinion there is usually no need to use more advanced HAR options, including computing a wavefunction for a cluster of molecules, when working with routine X-ray diffraction datasets. This issue was also discussed in some recent studies^43,50–52^

### 2.3. Conformational analysis

**Fig. 3:**
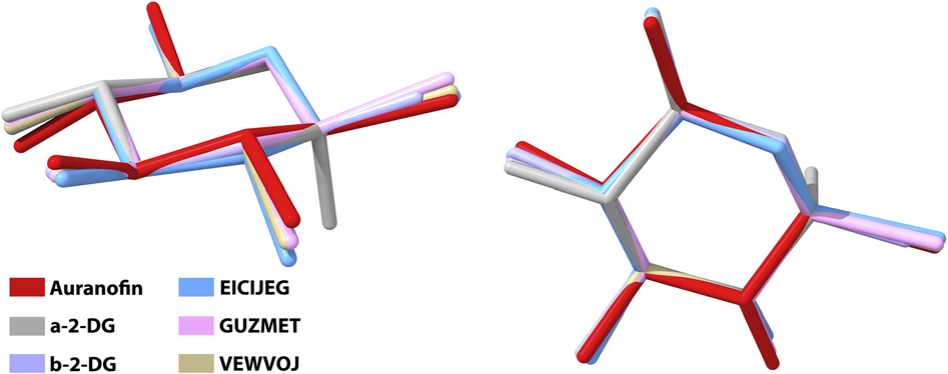
Structural alignment of several glucose–gold complexes compared to AF. Hydrogen atoms and substituents attached to the pyranose rings were omitted for the sake of clarity.

A convenient way to describe the conformation of carbohydrates in their cyclic forms is to use Cremer–Pople (CP) parameters, which are generalized ring-puckering coordinates in a spherical coordinate system.^53^ The CP parameters allow the ring puckering to be defined quantitatively. We compare the conformation of the pyranose ring in AF, both anomers of 2-deoxyglucose and selected complex compounds displaying structural similarity to AF; their names refer to given CCDC entries (Fig. 3 and Table S10†). In each of these structures, the pyranose ring adopts the chair ^4^C_1_ conformer, which is prevalent for most pyranoses either in solid state or aqueous solution. Only slight deviations from the ideal ^4^C_1_ conformation (*θ* = 0°) were observed in the case of auranofin and ECIJEG (*θ* = 11° for both compounds). These values of *θ* may result from additional strain imposed on the ring by the hydroxyl-protecting group attached to the pyranose moiety.

### 2.4. Intermolecular interactions

**Fig. 4:**
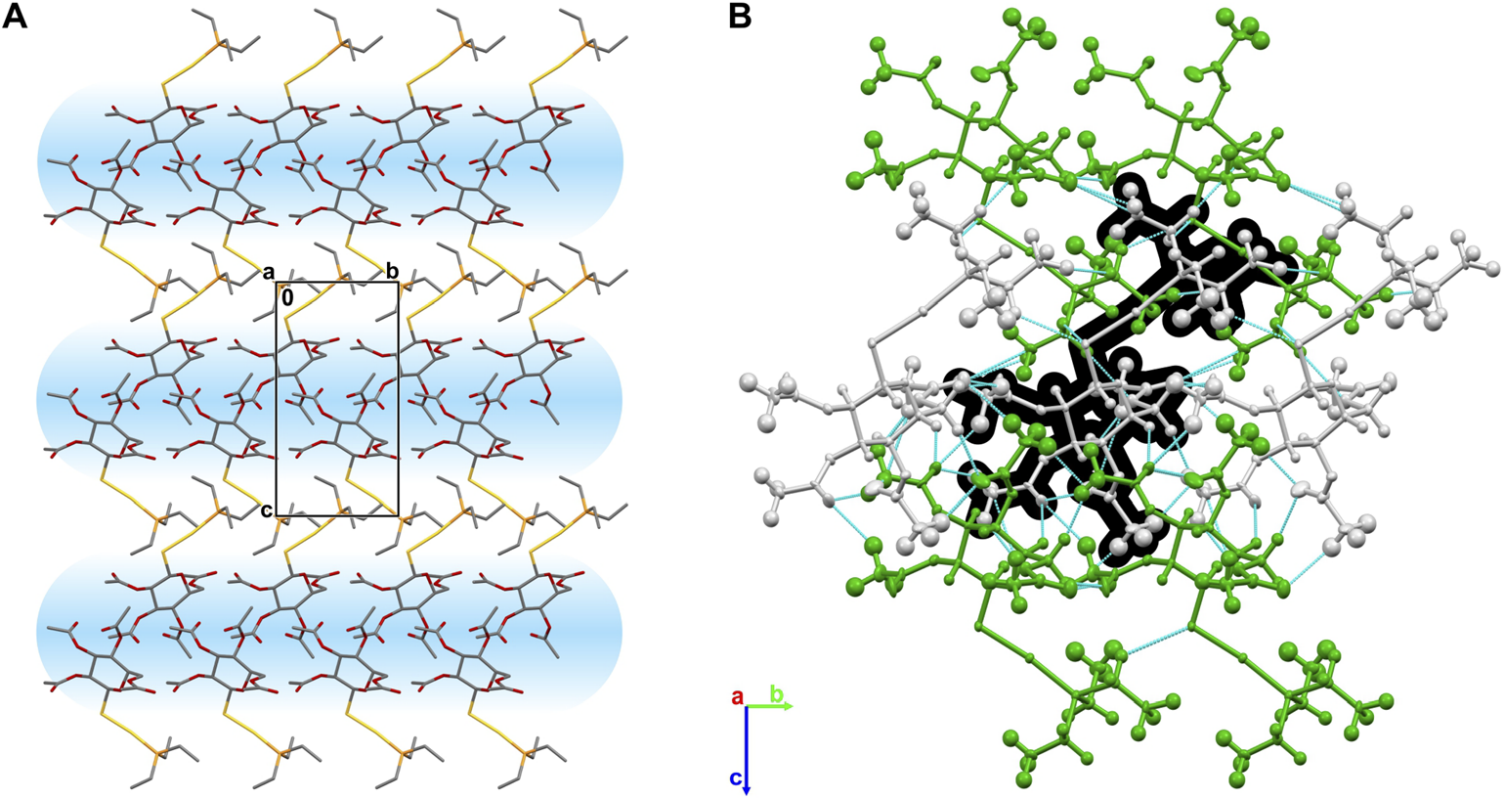
(A) The bilayer ABAB architecture of the AF crystal, with a layer composed of the head-to-head positioned pyranose rings displayed as blue strips. Hydrogens atoms were removed for the sake of clarity. (B) Molecules of AF coloured according to different symmetry codes. Intermolecular contacts are displayed as cyan lines. One molecule of AF is highlighted in black for better visibility.

The investigated AF crystal is composed of bilayers (Fig. 4) in which one layer consists of the head-to-head positioned pyranose rings (1; 1 − *x*, ½ + *y*, 1 − *z* symmetry codes), while the second layer consists of the phosphines (1; −*x*, −½ + *y*, −*z* symmetry codes). Contrary to the crystal structures of carbohydrates possessing free OH groups, in the AF crystals, there is a lack of any strong hydrogen bonds (HB), which significantly impacts the supramolecular architecture and stability of the system. The “pyranose layer” is mostly stabilised by weak HBs and the O–H⋯O interactions as well as the net of dispersive H⋯H contacts. The main intermolecular contacts in the crystal structure are four weak HBs: C–H⋯O *d*(D⋯A) = 3.43 Å; ∠(D–H⋯A) = 174.8°, C–H⋯O *d*(D⋯A) = 3.23 Å; ∠(D–H⋯A) = 143.1.0°, *d*(D⋯A) = 3.34 Å; ∠(D–H⋯A) = 140.5° and C–H⋯O *d*(D⋯A) = 3.39 Å; ∠(D–H⋯A) = 134.8° between O27–H22C, O27–H26A, O32–H26B and O23–H18A respectively. Other O⋯H interactions are of minor importance since they are not likely to form any HB. There are also some C–H⋯Au interatomic contacts present in the AF crystal: C–H⋯Au *d*(H⋯Au) ∈ [2.61, 3.36 ] Å; ∠(C–H⋯Au) ∈ [102.2, 167.1]°. These contact may form weak HBs involving the gold atom, which is further supported by computational analysis of electron density (see Paragraph 2.5 for the details). Despite the substitution of aliphatic phosphine with N-heterocyclic carbene ligands, these related gold complexes of acetylated pyranoses (Fig. S3†) maintain similar molecular architectures, with crystal packing primarily stabilised by weak hydrogen bonds between pyranose rings. Intermolecular interactions between ligand fragments occur predominantly through H⋯H contacts, with molecular geometry playing a minor role in crystal packing. In contrast, the parent glucopyranose and related carbohydrates form dense three-dimensional structures stabilised by extensive networks of strong O–H⋯O hydrogen bonds, resulting in rigid frameworks with higher crystallographic symmetry.^54,55^

**Fig. 5:**
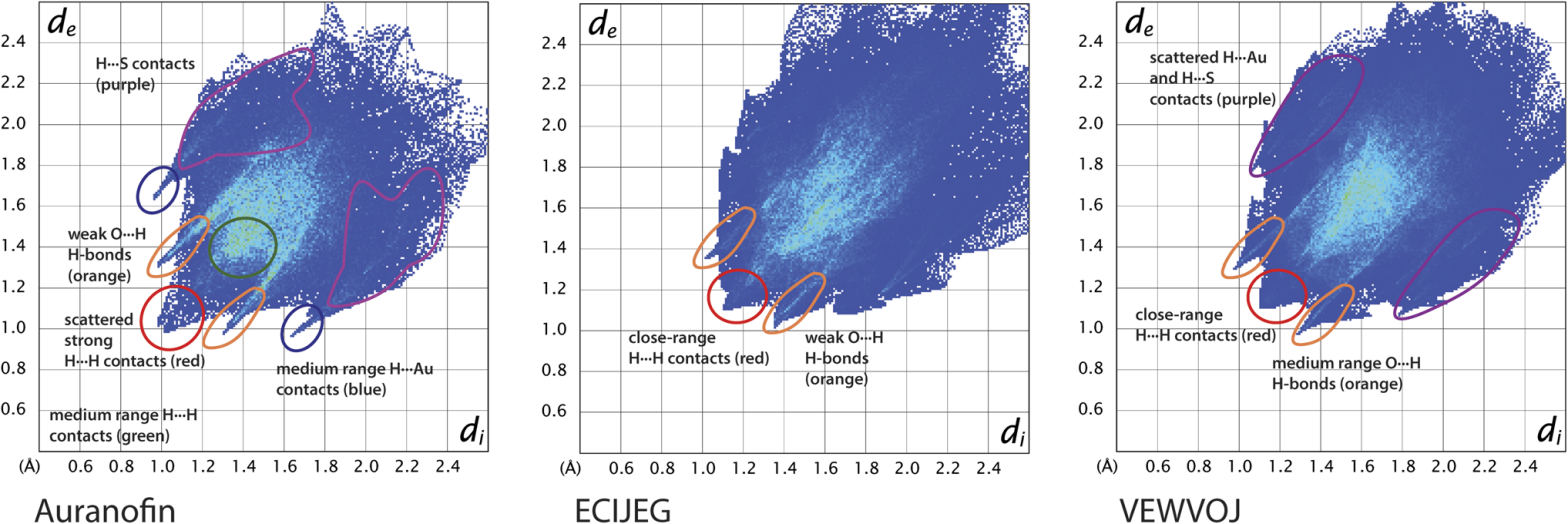
Two-dimensional fingerprint plots for the analysed crystals with indicated localisation of selected atom-atom interactions.

Hirshfeld surface (HS) in crystals represents equal electron density contributions from a specific molecule (promolecule) and its neighbouring molecules (procrystal). The HS analysis quantitatively depicts interatomic contacts within the crystal structure, showing intermolecular interactions. Parameters *d*_i_ and *d*_e_ denote the minimum distance from a point on the HS to an atom nucleus inside or outside the surface (Fig. S4†). These parameters can be visualised on fingerprint plots, which map the relation of *d*_i_ to *d*_e_ on the HS, allowing to conveniently underscore differences in the examined crystals. The fingerprints for AF and two related compounds are depicted in Fig. 5, and the contributions of each type of interatomic contacts are summarised in Table 1.

**Table 1:** Contribution of interatomic contacts to the overall Hirshfeld surface for chosen compounds expressed in percent [%]

Type of contact	Auranofin	Optimised auranofin	ECIJEG	VEWVOJ
Au⋯H	3.7	4.4	0.3	0.9
S⋯H	5.6	5.9	7.5	5.0
O⋯H	29.6	29.1	20.1	20.0
N⋯H	na	na	1.0	0.0
C⋯H	1.6	1.6	3.8	7.9
H⋯H	58.7	58.1	65.7	65.7
Au⋯O	0.1	0.2	0.0	0.0
O⋯O	0.4	0.5	1.0	0.3
C⋯O	0.2	0.1	0.8	0.0
C⋯S	0.0	0.0	0.0	0.2

The most important interatomic contacts in terms of both prevalence and interatomic interactions, are H⋯H and O⋯H; the latter forms small diagonal spikes typical for weak HB. The contributions of the scattered S⋯H and the C⋯H contacts are not essential, and these contacts are not involved in any meaningful interactions (Fig. S5†). The bulky ligands caged the gold atoms in each of the above crystals. They had limited intermolecular interactions with hydrogens, which is especially noticeable for N-carbene ligands tightly enclosing the gold atom. Interestingly, there are some differences between the AF fingerprints before and after geometry optimisation *via* periodic DFT calculations (Fig. S5†). The noticeable distinction is the presence of shorter Au⋯H interatomic contacts in the optimised structure, which are not present in the native crystal (Fig. S4†). This finding suggests that the gold atoms may be involved in true intermolecular interactions instead of being only artefacts caused by crystal packing.

### 2.5. Energetic features and charge density analysis

The total cohesive energies of the crystal lattice for AF and some other carbohydrates are presented in Table 2. The cohesive energy of the AF crystal appears higher than that of previously studied deoxy- or halogenated glucose derivatives, approaching values observed for disaccharides such as sucrose. However, direct energetic comparisons between these systems require careful consideration due to significant differences in molecular size and shape of the lattice constituents. In molecular crystals, where cohesive energy represents the sum of all intermolecular interactions, the molecular geometry and volume significantly influence crystal stability. For instance, a low value of the cohesive energy may not indicate high stability if the attractive interactions are “smeared” over the large molecular surface or are not involved in any vital, localised interactions. In order to compare the energetic features of AF crystal with some related compounds in a less biased manner, we propose following parameters: Hirshfeld surface area (HS area) and Hirshfeld surface volume (HS volume), which are the ratio of cohesive energy to either HS area or HS volume respectively. The first parameter, HS surface energy, is more relevant since it shows the relative strength of attractive interaction between neighbouring promolecules in the crystal and, therefore can be applied to compare the stability of a series of different crystals. A comparison of HS surface energies in Table 2 indicates much lower stability of AF crystal in relation to other compounds. They all possess free OH groups involved in forming strong H-bonds, which contribute much more to crystal stability than weak H-bonds and dispersive interactions in AF crystal.

**Table 2:** Cohesive energies and their relation to the geometry of HS for AF and several other carbohydrates. Cohesive energies for the other compounds are taken from the literature^56,57^

Compound^a^	Cohesive energy (kJ mol^−1^)	HS area (Å^2^)	HS volume (Å^3^)	HS surface energy (kJ mol^−1^ Å^−2^)	HS energy density (kJ mol^−1^ Å^−3^)
Auranofin	**−285.06**	551.83	623.22	**0.52**	**0.46**
α-2-DG	−197.10	179.93	176.94	1.10	1.12
β-2-DG	−215.00	179.93	176.95	1.19	1.22
Sucrose	−307.26	308.68	352.87	1.00	0.87
β-2-FG	−217.56	187.40	182.46	1.16	1.19
β-2-FM	−240.25	180.85	178.81	1.33	1.34
β-2-CG	−208.16	196.40	192.84	1.06	1.08
β-2-CM	−202.83	198.24	195.61	1.02	1.04
β-2-IG	−199.70	204.51	205.81	0.98	0.97
4-BS	−338.14	319.38	365.92	1.06	0.92

a Compound abbreviations: 2-deoxy-2-fluoro-d-glucopyranose (2-FG), 2-deoxy-2-fluoro-d-mannopyranose (2-FM), 2-chloro-2-deoxy-d-glucopyranose (2-CG), 2-chloro-2-deoxy-d-mannopyranose (2-CM), 2-deoxy-2-iodo-d-glucopyranose (2-IG), β-d-fructofuranosyl 4-bromo-4-deoxy-α-d-glucopyranoside (4-BS).

**Fig. 6:**
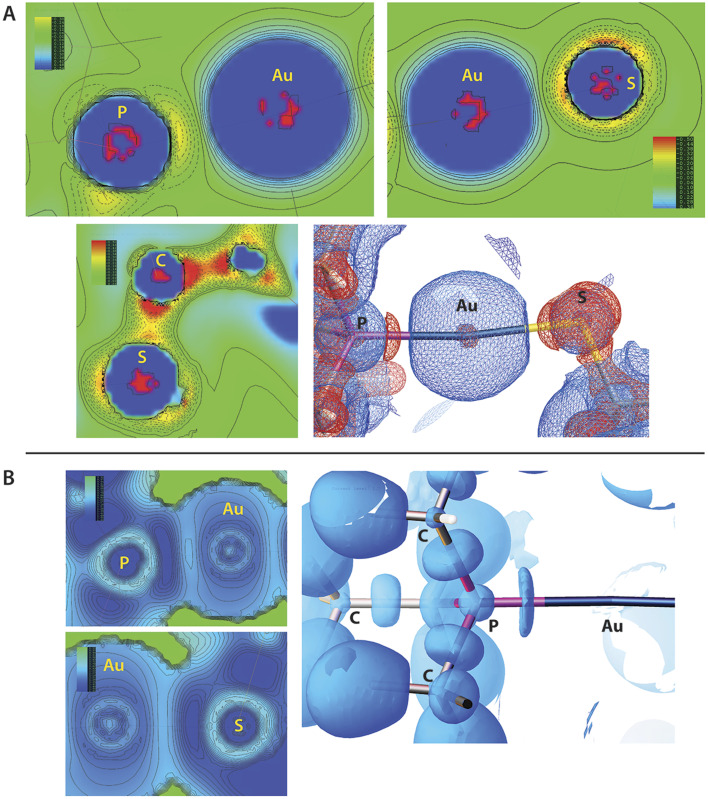
Charge-density analysis. (A) The contour representation of ∇^2^*ρ*(*r*)for selected fragments of auranofin molecule in the crystal structure and 3D representation of ∇^2^ for the P–Au–S bonds. The blue colour represents charge depletion (CD) while the red colour represents charge concentration (CC). Contour levels are set to 0.2 *e* A^−5^ for negative and positive charges in the 3D representation. (B) Contour representation of ELI-D for selected fragments of auranofin molecule and 3D representation ELI-D for P–Au–S bonds. The contour level is set to 1.7 *Y*_D_ (a dimensionless value) in the 3D representation.

We also performed a simple qualitative analysis of the charge-density properties of AF molecules in the crystal, similar to our earlier studies on brominated sugars.^58^ Electronic properties, *i.e.* electron density (ED), electrostatic potential (ESP), Laplacian of ED, ∇^2^*ρ*(*r*), and electron localizability index (ELI-D),^59^ were calculated using previously computed wavefunction for the experimental HAR-refined structure. Since both ∇^2^*ρ*(*r*) and (ELI-D) are related to ED, they can be directly applied to experimental data in X-ray crystallography. After calculations, both ED and its relatives were then transferred for the procrystal using appropriate symmetry operations (Fig. 6). Computed values of ESP were mapped onto the ED isosurface to create a mapped electrostatic potential (MEP), and the red-white-blue gradient represents the highest to lowest ESP values (Fig. S6†). Owing to the selected level of ED surface, located near nuclei the MEP values are positive everywhere. Overall, the MEP is dominated by Au and S atoms with the highest values, while methyl groups are less positive. Spots of more positive MEP computed for an AF dimer confirm the presence of C–H⋯O H-bonds (contour 0.03 *e* A^−3^) and also indicate possible sites of weak dispersive H⋯H interactions (contour 0.02 *e* A^−3^), which are both prevalent in the crystal structure. ∇^2^*ρ*(*r*) shows charge concentration (CC) regions slightly above the atomic surface of S atom as well as indicate covalent bonds in the molecule. In the P–Au–S region charge is depleted near the Au atom and the bonding contribution from both S and P is visible. The flattened shape of the CC region between P and Au atoms is typical for a coordinate bond, where the electron pair is donated by phosphorus. Analysis of ELI-D distribution around phosphorus atom shows typical C–P covalent bonds, with nearly symmetrical and oval ELI-D basins whereas coordinate P–Au bond have a basin flattened along the bond axis due to σ-coordination. Additionally, distribution of electron localization function (ELF)^60^ shows very similar pattern (Fig. S7†) as well as deformed region around the sulphur atom bonded to Au.

**Fig. 7:**
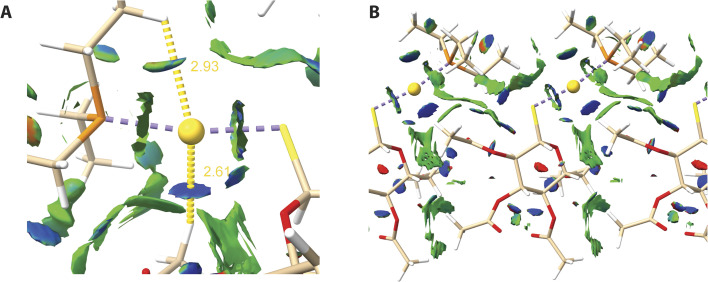
NCI isosurfaces (RDG value 0.6) for the AF crystal over the range of (−0.05 < sign(*λ*_2_)*ρ* < 0.05). Noncovalent interactions are coloured using RBG gradient where blue, green and red indicates strong positive, weak positive and destabilising interactions, respectively. (A) Interactions around gold atom. Yellow dot lines indicate distances between Au and near hydrogen atoms. (B) Noncovalent interactions around an AF molecule in a monolayer.

Interestingly, some recent studies have provided evidence for Au⋯H–C hydrogen bonding interactions in gold(i) complexes.^61,62^ Application of non-covalent interaction index (NCI), a visualization tool based on the Reduced Density Gradient (RDG) indicates that coordinated Au atom is likely to form H-bonds with adjacent hydrogens in the AF crystal (Fig. 7A). The Au⋯H–C distances are relatively short (2.61 and 2.93 Å) and the latter distance is shorter than the sum of the van der Waals (vdW) radii for gold and hydrogen (2.86 Å). The presence of a negative RDG region along the Au⋯H axis further supports this claim. Apart from the region around the Au coordination sphere, NCI indicates mostly vdW and weak H-bonds as stabilising interactions between AF molecules (Fig. 7B). Some dispersive contribution is also present in Au–S and Au–P covalent bonds, which is visible on NCI plots as rings around the bond axis.

### 2.6. Thermochemistry

**Fig. 8:**
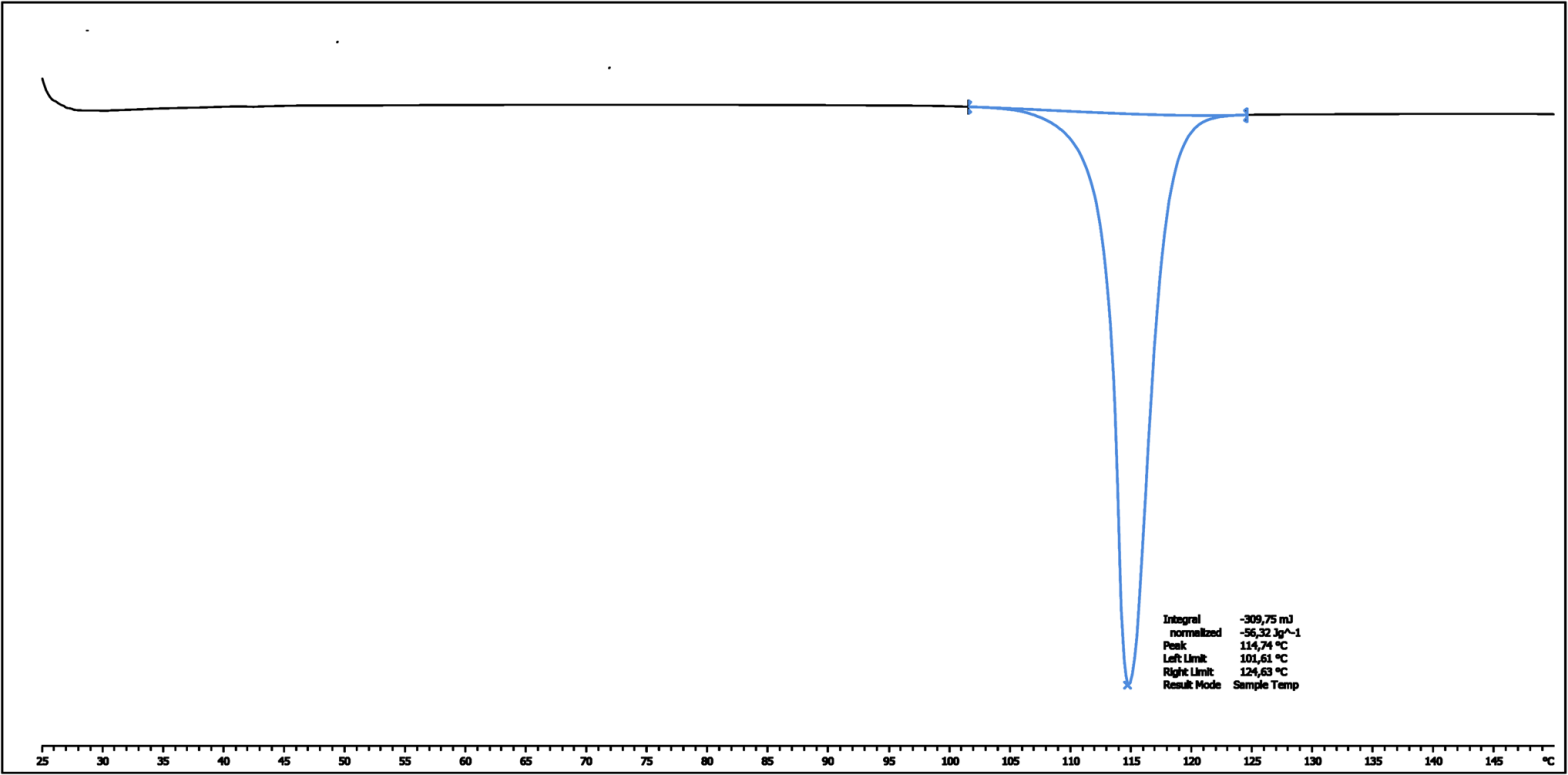
DSC melting thermogram for auranofin crystals measured for heat rating of 20 K min^−1^.

In search of any experimental evidence of the elusive AF polymorph, we used differential scanning calorimetry (DSC) coupled with thermal gravimetric analysis (TGA) to determine specific thermodynamic parameters and compare them with the existing literature.^35^ The DSC thermogram of crystalline AF (Fig. 8) exhibits a single melting endotherm, indicating phase purity without evidence of polymorphic contamination or solid-state phase transitions. The thermal profile remained consistent across multiple heating rates, with complementary TGA-DSC measurements confirming the absence of additional thermal events in the solid state (Fig. S8†). The overall shape of the DSC thermogram and melting temperature are virtually the same as reported previously (*T*_m_ 387.9 K in our study to 389 K in the earlier study). A similar situation is observed for enthalpy of fusion (Δ*H*_f_), in our study, the Δ*H*_f_ value is 38.21 kJ mol^−1^ while the previously reported value is 37.81 kJ mol^−1^. Given that 24.48 kJ mol^−1^, is expected for the B polymorph, the contamination by the second polymorph is expected to decrease rather than increase Δ*H*_f_. Small discrepancies in *T*_m_ and Δ*H*_f_ most likely are connected to the different equipment and possibly changes in the amount of trace water presented in the different samples. These findings confirm that our crystalline samples comprise exclusively the canonical A polymorph, which maintains phase stability without evidence of solid-state transformations throughout the studied temperature range.

### 2.7. Interatomic interactions of Au(i) in crystal state and protein complexes

In medicinal applications, organogold compounds serve as molecular carriers for biologically active Au(i) cations, with their therapeutic efficacy dependent on stability under physiological conditions. Consequently, we investigated structural variations in the Au(i) coordination sphere across different crystal forms and protein binding sites to better understand the molecular basis of their biological activity. To perform these comparisons, we analysed the Hirshfeld surfaces for gold atom in selected organic compounds and Au^+^ cation complexed by the protein (Fig. S9 and S10†). Despite the different chemical environments of the Au(i) atom in AF and triphenylphosphine, the discrepancies in HS are minimal. The coordination sphere in AF is slightly tighter than in the latter compound, and the only significant difference is the presence of relatively short Au⋯H interatomic contacts in the case of AF (Fig. S10†). Interestingly, such contacts are observed in coordination spheres of at least two Au-protein complexes. CueR is a bacterial protein with zeptomolar (10^−21^ molar) affinities for Cu^+^ and some other monovalent metal cations.^63^ A comparison between the binding of several different cations (Au^+^, Ag^+^, and Cu^+^) by CueR protein shows that other cations than Au^+^ do not tend to form closer M⋯H contacts.^64^ This findings may support the evidence of Au^+^ cation higher capacity (compared to some other M^+^ cations) to form weak HB within its coordination sphere. Comparative analysis of Hirshfeld surfaces demonstrates conserved Au(i) coordination geometry between molecular crystals and the Au–CueR complex, correlating with CueR's exceptional gold-binding affinity. Similar coordination environments are observed in glutathione reductase–Au(i) complexes, indicating favourable binding interactions and complex stability. Although precise determination of Au(i) binding constants to its molecular targets remains challenging, multiple studies suggest high-affinity interactions and complex stability both *in vitro* and in cellular environments.^65^ In certain Au–protein complexes, including thioredoxin reductase (Fig. S10C†), the exposed nature of binding sites limits reliable Hirshfeld surface analysis due to potential distortions. While crystallographic structures lack many coordinating water molecules present in solution, the Au(i) ions remain tightly bound despite solvent exposure, effectively inhibiting protein function.

## 3. Conclusions

We carried out a structural characterisation of AF using modern tools of X-ray crystallography and compared our findings to selected previously studied organogold compounds. In this example, the application of HAR only slightly improved the quality of the refinement providing somewhat better positions of hydrogen atoms. Additionally, we used molecular wavefunctions computed for AF structure for a simple charge-density analysis. All studied crystals display similar structural features and patterns of intermolecular interactions. The crystal structure of AF is layered and stabilised by many weak H-bonds and dispersive interactions including C–H⋯Au bonds, which is reflected in low HS energy values compared to glucose derivatives possessing free hydroxyl groups. Importantly, our studies indicate that the previously described polymorphic form of AF cannot be prepared using the methods mentioned in the literature. Despite testing different solvents, temperatures, and solubilisation methods, we could not obtain this polymorph. These findings may have further ramifications for the pharmaceutical research involving organogold compounds, which often display limited capability to be solved in aqueous solutions, potentially resulting in their limited bioavailability.

## 4. Materials and methods

### 4.1. Crystallization and inspection of crystal morphology

Auranofin was crystallized by slow evaporation from ethanol at ambient temperature. The resulting colourless, acicular crystals arranged in radial clusters exhibited a diffraction limit of 0.7 Å. Multiple crystallization attempts to obtain the B polymorph were performed using previously reported conditions (cyclohexane/ethyl acetate mixtures) and modified protocols including various non-polar solvents at temperatures ranging from 4–25 °C, but exclusively yielded the canonical A form. Obtained crystals of AF were studied using an optical microscope (Discovery. V8, Carl Zeiss) with a polarization filter. Photographs were taken using an external digital camera and processed in *Photoshop CS6* (Adobe Inc.) to adjust gamma and contrast levels for better visibility.

### 4.2. X-ray data collection and refinement

A good quality single crystal of auranofin was selected for the X-ray diffraction experiment at *T* = 100(2) K. Data were collected on the Agilent Technologies SuperNova Dual Source diffractometer using CuKα radiation (*λ* = 1.54184 Å) and CrysAlis Pro software (CrysAlis Pro, Oxford Diffraction/Agilent Technologies UK Ltd, Yarnton, England). Analytical, numerical absorption correction using a multifaceted crystal model, implemented in SCALE3 ABSPACK scaling algorithm was applied.^66^ The initial model of the structure was solved with direct methods followed by successive least-square refinement based on the full-matrix least-squares method on *F*^2^ using the *SHELX* package.^42^ Hydrogen atoms were located from the Fourier difference electron density map and refined with *U*_iso_(H) = 1.5O_eq_(O). The final aspherical structural refinement was carried out using *olex.refine* program with *NoSpherA2* functionalities implemented in *Olex2-1.5* software.^43^*ORCA 5.0* package^67^ was applied to compute a molecular wavefunction for either a single molecule or a trimer of molecules in the crystal at the PBE/x2c-TZVPP (x2c-TZVP for the cluster of molecules)^44,68^ level of theory using Douglas–Kroll–Hess (DHK2) relativistic correction.^47,69^ Calculations were performed on DefGrid2 integration grid. Aspherical scattering factors were calculated at a high level of integration precision. Hydrogen atoms were refined isotropically and all atoms were constrained to distances derived from neutron diffraction. Molecular interactions were identified using the *PLATON* program.^70^ All graphical representations of the crystal structures and molecular interactions were prepared using *Mercury*, *Olex2*, and *ChimeraX* programs.^46,71,72^ The final .cif file has been deposited in CSD database (entry 2402671).†

### 4.3. Cohesive energy and Hirshfeld surface calculations

Optimization of the molecular geometry of the auranofin crystal at fixed lattice parameters was performed using *Crystal17* with the DFT method and B3LYP functional.^73,74^ We applied 6-31 G(d,p) basis set for light elements^75^ (H, C, O, P, S) and pob_TZVP_rev2 for Au.^76^ The crystal lattice energy was computed with Grimme dispersion and BSSE corrections in accordance with the manual provided by the software developer (Crystal Solutions).^77^ Hirshfeld surfaces and two-dimensional fingerprint plots were calculated using *Crystal Explorer 21*, and the atomic coordinates used in the calculations were either taken from the structure optimised in *Crystal17* (auranofin) or structures from the CCDC database (other compounds) with their geometry corrected in *Crystal Explorer*.^78^

### 4.4. Thermochemistry

A series of DSC measurements were performed using the Mettler–Toledo DSC1 STAR system at a heating rate of 5, 10, and 20 K min^−1^ under a dry N_2_ atmosphere and at a constant flow (50 mL min^−1^) over a range of temperature from 298 K to 423 K. The total weight of each sample was accurately weighted into a standard 40 μL aluminium crucible using Mettler–Toledo XS105 DualRange balance. Additionally, the TGA/DSC measurement using the TGA/DSC module of the above-mentioned Mettler–Toledo system was also undertaken. The experiment was also performed under a dry N_2_ atmosphere (constant flow, 50 mL min^−1^) over a temperature range from 298 to 887 K. In this case, the sample was weighted into a standard 70 μL alumina crucible using Mettler–Toledo XS105 DualRange balance. Obtained data were analysed using the STARe software provided by Mettler–Toledo.

### 4.5. Charge-density calculations

Molecular wavefunction was computed in *ORCA 5.0* package^67^ at the R2SCAN/x2c-TZPP level of theory^68,79^ using DHK2 relativistic correction.^47^ All electron density maps and their derivatives, *i.e.*, Laplacian of electron density and ELI-D, were calculated in *NoSpherA2*. The molecular wavefunction for ESP calculations was computed in ORCA. 5.0 at the R2SCAN/x2c-SVP level of theory using DHK2 relativistic correction, and ESP map was also computed in *NoSpherA2*. All maps were rendered and visualised in *Olex2*. The molecular wavefunction of AF trimer, after periodic geometry optimization, for NCI and ELF analysis was computed in ORCA. 5.0 at the wB97X-D4/x2c-TZVPall level of theory^49,80^ using SEQCROW bundle in ChimeraX.^81^ All calculations were carried out on DefGrid2 integration grid. The ELF and NCI were computed using Multiwfn 3.8 (ref. 82) program and also visualised in ChimeraX.

## Data availability

Detailed crystallographic data are available in ESI in the following file: ESI.† A complete .cif file (including *hkl* parameters) of the auranofin structure is available in ESI† in the following file: Auranofin.cif. Both files were uploaded during the submission process. The .cif file is also deposited in the Cambridge Structural Database, Deposition Number: 2402671.†

## Conflicts of interest

There are no conflicts to declare.

## Supplementary Material

RA-015-D5RA00196J-s001

RA-015-D5RA00196J-s002
