# Supplementary material for: Crystal structure and polymorphic forms of auranofin revisited
Source: RSC Adv. 2025 Apr 3;15(13):10378–89. doi: 10.1039/d5ra00196j (PMC11967170; doi:10.1039/d5ra00196j)
Supplement: RA-015-D5RA00196J-s001 [file RA-015-D5RA00196J-s001.pdf]

Supplementary Information (SI) for RSC Advances.
This journal is © The Royal Society of Chemistry 2025

**Supplementary:**

All crystallographic data are provided for the model refined using HAR and a small cluster of molecules to compute molecular wavefunction.

**Table S1.** Crystal data and structure refinement for investigated compound.

| | |
|---|---|
| Empirical formula | $C_{20}H_{34}AuO_9PS$ |
| Formula weight | 678.494 |
| Temperature/K | 100(2) |
| Crystal system | monoclinic |
| Space group | $P2_1$ |
| $a$/Å | 10.0684(4) |
| $b$/Å | 8.1314(3) |
| $c$/Å | 16.1310(6) |
| $\alpha$/° | 90 |
| $\beta$/° | 106.247(4) |
| $\gamma$/° | 90 |
| Volume/Å$^3$ | 1267.91(8) |
| $Z$ | 2 |
| $\rho_{calc}$g/cm$^3$ | 1.777 |
| $\mu$/mm$^{-1}$ | 12.487 |
| $F$(000) | 667.8 |
| Crystal size/mm$^3$ | 0.25 × 0.06 × 0.04 |
| Radiation | Cu $K\alpha$ ($\lambda$ = 1.54184) |
| 2$\Theta$ range for data collection/° | 5.7 to 134.02 |
| Index ranges | $-12 \leq h \leq 7$, $-9 \leq k \leq 9$, $-18 \leq l \leq 19$ |
| Reflections collected | 8687 |
| Independent reflections | 4525 [$R_{int}$ = 0.0332, $R_{sigma}$ = 0.0421] |
| Data/restraints/parameters | 4525/1/296 |
| Goodness-of-fit on $F^2$ | 1.032 |
| Final $R$ indexes [$I$>=2σ ($I$)] | $R_1$ = 0.0273, w$R_2$ = 0.0694 |
| Final $R$ indexes [all data] | $R_1$ = 0.0283, w$R_2$ = 0.0704 |
| Largest diff. peak/hole / e Å$^{-3}$ | 0.95/-1.23 |
| Flack parameter | -0.049(6) |

**Table S2.** Fractional Atomic Coordinates (×10$^4$) and Equivalent Isotropic Displacement Parameters (Å$^2$×10$^3$). $U_{eq}$ is defined as 1/3 of the trace of the orthogonalised $U_{IJ}$ tensor.

| **Atom** | ***x*** | ***y*** | ***z*** | **U(eq)** |
|---|---|---|---|---|
| **Atom** | ***x*** | ***y*** | ***z*** | **U(eq)** |
| Au1 | 3171.60(17) | 3101.06(6) | 743.72(11) | 15.28(7) |
| C1 | 4677(6) | 1284(6) | 2580(4) | 16.3(11) |
| C2 | 5303(6) | -237(6) | 3087(4) | 14.2(11) |
| C3 | 6140(5) | 222(6) | 3994(3) | 12.6(10) |
| C4 | 7230(6) | 1500(6) | 3951(3) | 15.6(11) |
| C5 | 6611(5) | 2901(7) | 3312(3) | 16.1(11) |
| C6 | 7739(6) | 3974(6) | 3131(4) | 19.7(12) |
| C7 | 4273(7) | -2896(6) | 2801(4) | 24.0(14) |
| C8 | 6458(5) | -1947(8) | 5035(3) | 18.6(10) |
| C9 | 9037(7) | 1887(7) | 5273(4) | 28.7(14) |
| C10 | 9710(6) | 3576(6) | 2629(4) | 25.9(15) |
| C11 | 3050(7) | -3910(7) | 2843(5) | 36.2(16) |
| C12 | 7072(7) | -3645(6) | 5204(4) | 25.8(13) |
| C13 | 9466(7) | 2946(8) | 6064(4) | 36.7(15) |
| C14 | 10795(6) | 2353(7) | 2563(4) | 28.7(14) |
| C15 | 3669(6) | 7087(6) | 144(4) | 23.4(13) |
| C16 | 837(6) | 5894(6) | -352(4) | 23.6(12) |
| C17 | 2801(6) | 4738(6) | -1240(4) | 21.4(12) |

**Table S2.** Fractional Atomic Coordinates (×10$^{4}$) and Equivalent Isotropic Displacement Parameters (Å$^{2}$×10$^{3}$). $U_{eq}$ is defined as 1/3 of the trace of the orthogonalised $U_{IJ}$ tensor.

| **Atom** | ***x*** | ***y*** | ***z*** | **U(eq)** |
|---|---|---|---|---|
| C18 | 3273(6) | 8550(5) | -482(4) | 24.4(14) |
| C19 | 500(7) | 6292(7) | 493(4) | 38.2(17) |
| C20 | 2063(6) | 3154(8) | -1639(3) | 27.7(11) |
| O1 | 5826(4) | 2240(4) | 2506(2) | 15.9(8) |
| O2 | 4242(4) | -1381(4) | 3158(3) | 19.1(9) |
| O3 | 6849(4) | -1251(4) | 4385(3) | 18.0(9) |
| O4 | 7750(4) | 2280(4) | 4785(2) | 19.1(8) |
| O6 | 8734(4) | 2912(5) | 2924(3) | 25.9(9) |
| O7 | 5177(5) | -3353(4) | 2500(4) | 38.6(12) |
| O8 | 5705(5) | -1329(4) | 5401(3) | 31.0(11) |
| O9 | 9714(5) | 803(5) | 5093(4) | 48.0(15) |
| O10 | 9714(5) | 5009(4) | 2433(4) | 38.7(12) |
| P1 | 2630.5(14) | 5263.6(12) | -178.0(9) | 16.7(3) |

**Table S3**. Anisotropic Displacement Parameters (Å$^{2}$×10$^{3}$). The Anisotropic displacement factor exponent takes the form: -2π$^{2}$[h$^{2}$a*$^{2}$$U_{11}$+2hka*b*$U_{12}$+…].

| **Atom** | $U_{11}$ | $U_{22}$ | $U_{33}$ | $U_{12}$ | $U_{13}$ | $U_{23}$ |
|---|---|---|---|---|---|---|
| Au1 | 17.68(11) | 13.51(11) | 14.11(10) | 0.11(12) | 3.57(7) | 1.25(12) |
| C1 | 21(3) | 9(3) | 19(3) | -4(2) | 5(2) | 2(2) |
| C2 | 18(3) | 9(3) | 16(3) | -1(2) | 4(2) | 2(2) |
| C3 | 17(3) | 11(3) | 11(2) | -3(2) | 6(2) | 1(2) |
| C4 | 15(3) | 17(3) | 14(3) | 2(2) | 2(2) | -3(2) |
| C5 | 23(2) | 11(3) | 14(2) | -1(3) | 3.7(18) | -1(2) |
| C6 | 20(3) | 16(3) | 23(3) | -2(2) | 5(2) | -1(2) |
| C7 | 31(4) | 10(3) | 29(3) | -5(3) | 5(3) | 1(3) |
| C8 | 27(2) | 18(2) | 11(2) | 1(4) | 6.1(18) | 5(4) |
| C9 | 30(3) | 27(3) | 24(3) | 7(3) | 0(3) | -8(3) |
| C10 | 24(3) | 22(4) | 33(3) | -4(2) | 10(3) | 3(2) |
| C11 | 35(4) | 27(4) | 44(4) | -17(3) | 7(3) | 6(3) |
| C12 | 34(3) | 19(3) | 24(3) | 1(3) | 9(3) | 6(2) |
| C13 | 42(3) | 39(4) | 20(3) | 5(4) | -7(2) | -3(4) |
| C14 | 18(3) | 39(4) | 30(3) | -2(3) | 9(3) | -4(3) |
| C15 | 27(3) | 21(3) | 21(3) | -2(2) | 4(2) | -4(2) |
| C16 | 22(3) | 25(3) | 22(3) | 1(2) | 2(2) | 2(3) |
| C17 | 27(3) | 18(3) | 22(3) | 0(2) | 12(3) | 1(2) |
| C18 | 32(3) | 13(3) | 27(3) | -5(2) | 7(3) | 0(2) |
| C19 | 40(4) | 43(4) | 35(4) | 22(3) | 17(3) | 3(3) |
| C20 | 40(3) | 27(3) | 19(2) | -2(5) | 14(2) | -6(4) |
| O1 | 23(2) | 12.6(19) | 14.4(18) | -2.1(15) | 9.0(16) | 1.3(15) |
| O2 | 20.4(19) | 18(2) | 18(2) | -3.1(15) | 3.9(16) | 4.0(15) |
| O3 | 19(2) | 16.9(19) | 20(2) | 4.1(14) | 8.0(16) | 4.8(15) |
| O4 | 20(2) | 20(2) | 16(2) | 2.4(16) | 2.1(16) | -2.0(16) |
| O6 | 26.9(18) | 25(3) | 31(2) | -3(2) | 17.7(16) | 1(2) |
| O7 | 40(3) | 14(2) | 63(4) | -1(2) | 16(3) | -11(2) |
| O8 | 49(3) | 25(2) | 27(2) | 8.7(19) | 24(2) | 5.8(17) |
| O9 | 29(3) | 61(4) | 43(3) | 12(3) | -6(2) | -16(3) |
| O10 | 39(3) | 31(3) | 53(3) | -1(2) | 24(2) | 2(2) |
| P1 | 19.5(7) | 13.5(7) | 16.1(7) | -0.3(5) | 3.5(5) | 1.7(5) |
| S1 | 21.7(6) | 11.7(6) | 17.0(6) | -2.0(5) | 1.5(5) | 0.6(5) |

**Table S4**. Bond Lengths.

| **Atom** | **Atom** | **Length/Å** | **Atom** | **Atom** | **Length/Å** |
|---|---|---|---|---|---|
| **Atom** | **Atom** | **Length/Å** | **Atom** | **Atom** | **Length/Å** |
| Au1 | P1 | 2.2686(12) | C7 | O7 | 1.204(8) |
| Au1 | S1 | 2.3004(11) | C8 | C12 | 1.506(8) |
| C1 | C2 | 1.520(7) | C8 | O3 | 1.343(6) |
| C1 | O1 | 1.426(6) | C8 | O8 | 1.195(7) |
| C1 | S1 | 1.806(6) | C9 | C13 | 1.499(8) |
| C2 | C3 | 1.516(7) | C9 | O4 | 1.353(8) |
| C2 | O2 | 1.444(6) | C9 | O9 | 1.198(7) |

**Table S4**. Bond Lengths.

| Atom | Atom | Length/Å | Atom | Atom | Length/Å |
|---|---|---|---|---|---|
| C3 | C4 | 1.526(7) | C10 | C14 | 1.503(8) |
| C3 | O3 | 1.445(6) | C10 | O6 | 1.321(7) |
| C4 | C5 | 1.547(7) | C10 | O10 | 1.208(6) |
| C4 | O4 | 1.446(6) | C15 | C18 | 1.540(7) |
| C5 | C6 | 1.524(7) | C15 | P1 | 1.805(5) |
| C5 | O1 | 1.423(6) | C16 | C19 | 1.528(8) |
| C6 | O6 | 1.431(6) | C16 | P1 | 1.822(6) |
| C7 | C11 | 1.500(9) | C17 | C20 | 1.536(8) |
| C7 | O2 | 1.364(6) | C17 | P1 | 1.820(6) |

**Table S5**. Bond Angles.

| Atom | Atom | Atom | Angle/° | Atom | Atom | Atom | Angle/° |
|---|---|---|---|---|---|---|---|
| S1 | Au1 | P1 | 172.73(4) | O8 | C8 | O3 | 125.0(6) |
| O1 | C1 | C2 | 105.3(4) | O4 | C9 | C13 | 111.1(5) |
| S1 | C1 | C2 | 110.2(3) | O9 | C9 | C13 | 125.5(6) |
| S1 | C1 | O1 | 111.6(4) | O9 | C9 | O4 | 123.4(6) |
| C3 | C2 | C1 | 110.7(4) | O6 | C10 | C14 | 112.6(4) |
| O2 | C2 | C1 | 111.2(4) | O10 | C10 | C14 | 124.3(6) |
| O2 | C2 | C3 | 107.8(4) | O10 | C10 | O6 | 123.1(6) |
| C4 | C3 | C2 | 109.7(4) | P1 | C15 | C18 | 115.0(4) |
| O3 | C3 | C2 | 106.9(4) | P1 | C16 | C19 | 112.3(4) |
| O3 | C3 | C4 | 108.1(4) | P1 | C17 | C20 | 115.4(4) |
| C5 | C4 | C3 | 111.8(4) | C5 | O1 | C1 | 112.7(4) |
| O4 | C4 | C3 | 109.5(4) | C7 | O2 | C2 | 116.3(4) |
| O4 | C4 | C5 | 105.7(4) | C8 | O3 | C3 | 118.3(4) |
| C6 | C5 | C4 | 111.5(4) | C9 | O4 | C4 | 118.4(4) |
| O1 | C5 | C4 | 110.3(4) | C10 | O6 | C6 | 118.5(4) |
| O1 | C5 | C6 | 107.1(4) | C15 | P1 | Au1 | 115.7(2) |
| O6 | C6 | C5 | 107.9(4) | C16 | P1 | Au1 | 111.41(19) |
| O2 | C7 | C11 | 111.2(6) | C16 | P1 | C15 | 106.4(3) |
| O7 | C7 | C11 | 124.8(5) | C17 | P1 | Au1 | 111.49(17) |
| O7 | C7 | O2 | 123.9(5) | C17 | P1 | C15 | 105.3(3) |
| O3 | C8 | C12 | 109.5(4) | C17 | P1 | C16 | 105.8(3) |
| O8 | C8 | C12 | 125.5(5) | C1 | S1 | Au1 | 105.25(15) |

**Table 6.** Torsion Angles.

| A | B | C | D | Angle/° | A | B | C | D | Angle/° |
|---|---|---|---|---|---|---|---|---|---|
| C1 | C2 | C3 | C4 | -55.2(5) | C3 | C4 | C5 | O1 | -49.4(4) |
| C1 | C2 | C3 | O3 | -172.1(4) | C3 | C4 | O4 | C9 | 106.6(5) |
| C1 | C2 | O2 | C7 | 118.8(5) | C3 | O3 | C8 | C12 | 166.7(5) |
| C1 | O1 | C5 | C4 | 62.0(4) | C3 | O3 | C8 | O8 | -11.1(5) |
| C1 | O1 | C5 | C6 | -176.5(4) | C4 | C5 | C6 | O6 | 49.1(4) |
| C2 | C3 | C4 | C5 | 46.8(4) | C4 | O4 | C9 | C13 | 173.5(5) |
| C2 | C3 | C4 | O4 | 163.6(4) | C4 | O4 | C9 | O9 | -8.1(5) |
| C2 | C3 | O3 | C8 | -111.3(4) | C5 | C6 | O6 | C10 | 171.0(4) |
| C2 | O2 | C7 | C11 | -174.7(5) | C6 | O6 | C10 | C14 | 171.6(5) |
| C2 | O2 | C7 | O7 | 6.0(6) | C6 | O6 | C10 | O10 | -8.7(6) |
| C3 | C4 | C5 | C6 | -168.3(4) | | | | | |

**Table 7.** Hydrogen Atom Coordinates (Å×$10^4$) and Isotropic Displacement Parameters (Å$^2$×$10^3$) for AF.

| Atom | *x* | *y* | *z* | U(eq) |
|---|---|---|---|---|
| H1 | 4090(6) | 1968(6) | 2933(4) | 19.6(13) |
| H2 | 5976(6) | -836(6) | 2760(4) | 17.0(13) |
| H3 | 5472(5) | 684(6) | 4363(3) | 15.1(12) |
| H4 | 8071(6) | 915(6) | 3767(3) | 18.8(14) |
| H5 | 5945(5) | 3655(7) | 3579(3) | 19.3(14) |
| H28a | 7295(6) | 4801(6) | 2592(4) | 23.6(14) |
| H28b | 8232(6) | 4714(6) | 3697(4) | 23.6(14) |
| H18a | 3120(30) | -4180(50) | 3507(5) | 54(2) |

**Table 7.** Hydrogen Atom Coordinates (Å×10[4]) and Isotropic Displacement Parameters (Å[2]×10[3]) for AF.

| **Atom** | ***x*** | ***y*** | ***z*** | **U(eq)** |
|---|---|---|---|---|
| H18b | 2113(8) | -3230(30) | 2560(30) | 54(2) |
| H18c | 3040(30) | -5040(30) | 2490(30) | 54(2) |
| H22a | 6440(30) | -4500(11) | 4754(19) | 39(2) |
| H22b | 8100(18) | -3639(14) | 5130(30) | 39(2) |
| H22c | 7120(40) | -4010(20) | 5853(10) | 39(2) |
| H26a | 9990(50) | 4020(30) | 5928(11) | 55(2) |
| H26b | 8563(9) | 3320(60) | 6250(20) | 55(2) |
| H26c | 10150(50) | 2260(20) | 6582(11) | 55(2) |
| H31a | 11660(20) | 2430(40) | 3132(14) | 43(2) |
| H31b | 10367(15) | 1131(8) | 2510(30) | 43(2) |
| H31c | 11130(40) | 2620(40) | 2000(17) | 43(2) |
| H10a | 4748(6) | 6772(6) | 215(4) | 28.1(16) |
| H10b | 3590(6) | 7473(6) | 776(4) | 28.1(16) |
| H12a | 157(6) | 4910(6) | -684(4) | 28.3(15) |
| H12b | 635(6) | 6977(6) | -768(4) | 28.3(15) |
| H14a | 3899(6) | 4617(6) | -1193(4) | 25.7(15) |
| H14b | 2397(6) | 5756(6) | -1679(4) | 25.7(15) |
| H11a | 3480(40) | 8245(19) | -1084(10) | 37(2) |
| H11b | 2189(11) | 8820(30) | -600(20) | 37(2) |
| H11c | 3870(30) | 9611(14) | -203(11) | 37(2) |
| H13a | -577(14) | 6610(60) | 359(6) | 57(3) |
| H13b | 720(50) | 5230(20) | 911(14) | 57(3) |
| H13c | 1130(40) | 7310(40) | 805(17) | 57(3) |
| H15a | 2300(40) | 2900(30) | -2236(14) | 41.5(16) |
| H15b | 2410(30) | 2145(13) | -1198(12) | 41.5(16) |
| H15c | 962(6) | 3310(20) | -1760(30) | 41.5(16) |

**Table S8.** Hydrogen-bond geometry in the crystal of **AF.**

| **D−H···A** | **d(D−H) [Å]** | **d(H···A) [Å]** | **d(D···A) [Å]** | **<D−H···A [ °]** |
|---|---|---|---|---|
| C11−H18A···O8$^{i}$ | 1.076(14) | 2.53(3) | 3.388(8) | 163(3) |
| C12−H22A···O8$^{i}$ | 1.08(2) | 2.58(3) | 3.465(8) | 140(2) |
| C12−H22B···O9$^{ii}$ | 1.08(2) | 2.37(2) | 3.431(9) | 168.5(15) |
| C13−H26A···O9$^{iii}$ | 1.07(4) | 2.28(3) | 3.229(8) | 146.8(19) |
| C13−H26C···O10$^{ii}$ | 1.08(3) | 2.403(18) | 3.338(8) | 144(3) |
| C6−H28A···O7$^{iv}$ | 1.091(8) | 2.578(8) | 3.308(7) | 123.6(5) |
| C6−H28B···O9$^{iii}$ | 1.090(8) | 2.571(8) | 3.590(8) | 155.1(6) |

Symmetry codes: (i) $-x + 1, y - 1, -z + 1$; (ii) $-x + 2, y - 1/2, -z + 1$; (iii) $-x + 2, y + 1/2, -z + 1$; (iv) $x, y + 1, z$.

**Table. S9**. Comparison of global refinement indicators in IAM and HAR methods.

| Quality indicator | IAM | HAR single | HAR cluster |
|---|---|---|---|
| $R_1$ | 2.78 | 2.73 | 2.73 |
| $wR_1$ | 7.11 | 7.00 | 7.00 |
| GooF | 1.064 | 1.039 | 1.038 |
| DFIX[a] | 34[b] | 18 | 19 |

[a]Number of H-X bonds with fixed length. [b]In the case of IAM refinement all H-X bond has fixed length imposed by default constraints for X-ray crystallography.

**Table S10**. Cremer-Pople parameters for crystal structures of **AF** and selected compounds.

| Compound | $q_2$ | $q_3$ | $Q$ | $\theta$ [°] | $\varphi_2$ [°] |
|---|---|---|---|---|---|
| auranofin | 0.113 | 0.557 | 0.588 | 11.0 | 56.0 |
| α-2-DG | 0.060 | 0.548 | 0.551 | 6.0 | 256.0 |
| β-2-DG | 0.038 | 0.562 | 0.563 | 3.9 | 350.4 |
| GUZMET | 0.041 | 0.600 | 0.601 | 3.8 | 345.0 |

| | | | | | |
|---|---|---|---|---|---|
| ECIJEG | 0.130 | 0.550 | 0.560 | 11.0 | 358.0 |
| VEWVOJ | 0.025 | 0.578 | 0.579 | 2.1 | 331.0 |

**Tab. S11**. Contribution of each type of interatomic contact to the overall Hirshfeld surface for other compounds mentioned in the publication expressed in percent [%].

| Type of contact | EKEVOI | UPELAF | UPIQUI |
|---|---|---|---|
| Au···H | 0.9 | 1.4 | 2.8 |
| S···H | 5.6 | 4.1 | 5.2 |
| O···H | 22.2 | 22.5 | 17.7 |
| N···H | 0 | 1.1 | 0.1 |
| C···H | 3.9 | 16.1 | 22.2 |
| H···H | 67.3 | 50.0 | 51.5 |
| Au···O | 0.0 | 0.0 | 0.0 |
| O···O | 0.0 | 0.5 | 0.0 |
| C···O | 0.2 | 0.5 | 0.2 |
| C···S | 0.0 | 0.8 | 0.0 |

**Table S12**. Enrichment ratios for selected interatomic contacts on HS

| Compound | Au-H | H-S | H-O | H-C | H-H |
|---|---|---|---|---|---|
| Auranofin | 1.24 | 1.22 | 1.27 | 1.14 | 0.94 |
| Optimised auranofin | 1.22 | 1.23 | 1.27 | 1.20 | 0.94 |
| ECIJEG | 1.23 | 1.08 | 1.23 | 1.01 | 0.99 |
| VEVWOY | 1.21 | 1.18 | 1.21 | 1.18 | 0.96 |

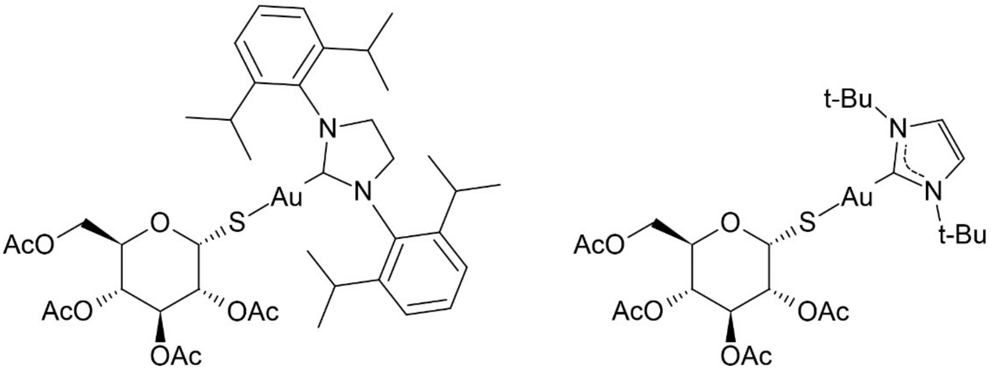


**Scheme S1**. Structural formulae of related compounds VEWVOJ (left) and ECIJEG (right).

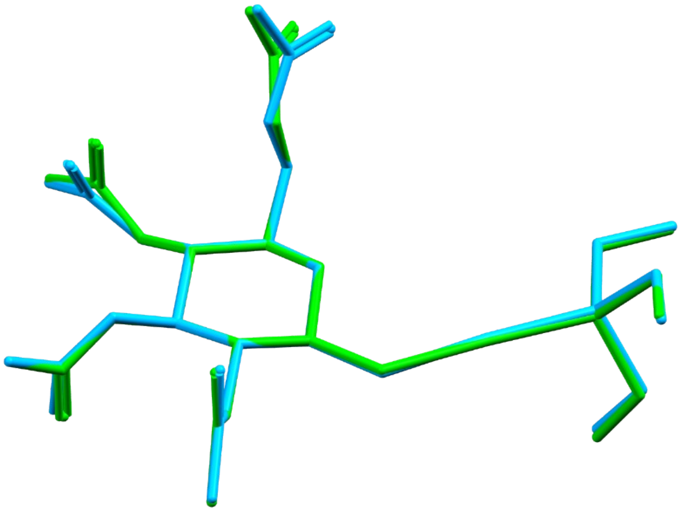

**Fig. S1**. Structural overlay of our auranofin structure (blue) with the existing old structure (green). Only non-hydrogen atoms are displayed.

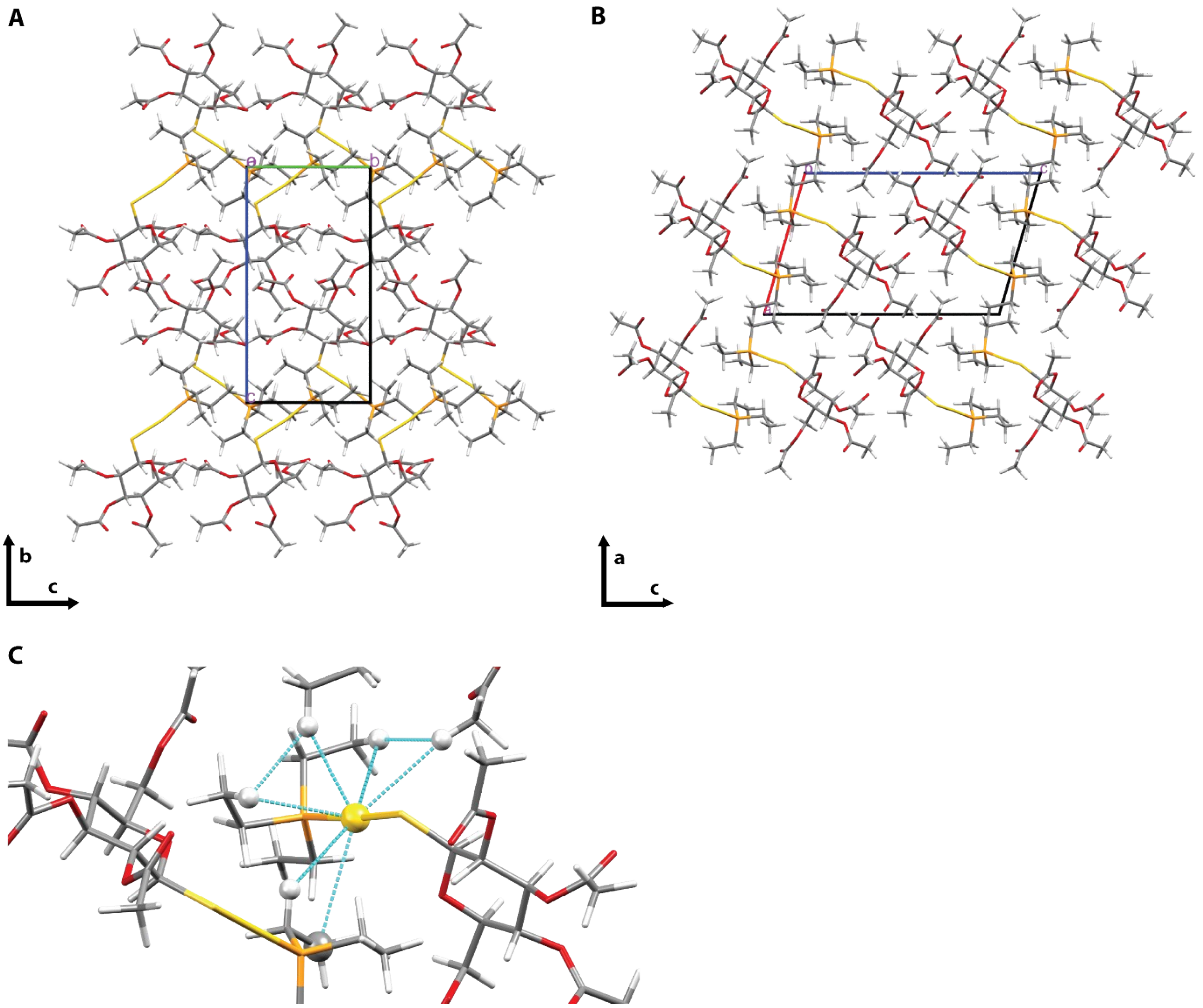


**Fig. S2**. The molecular packing of **AF** crystal along *a* and *b* directions (**A**, **B** panels respectively). **C**. Close interatomic contacts in the vicinity of Au atom, contacts are depicted as cyan dotted lines.

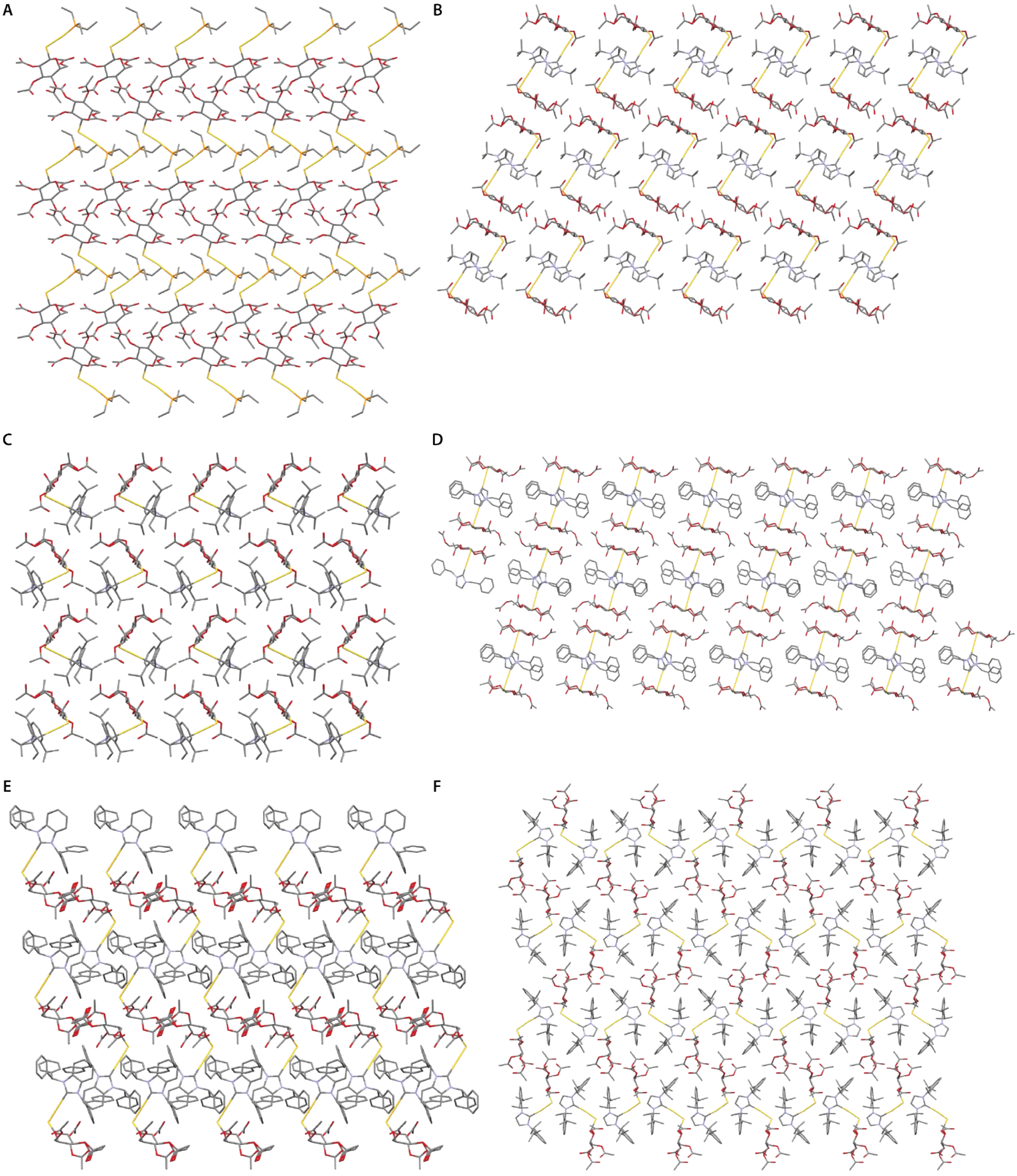


**Fig. S3**. Comparison between crystal structure of auranofin (**A**) and several related compounds: ECIJEG (**B**), EKEVOI (**C**), UPELAF (**D**), UPIQUI (**E**) and VEWVOJ (**F**) with adjusted orientation adjusted to highlight their layered composition.

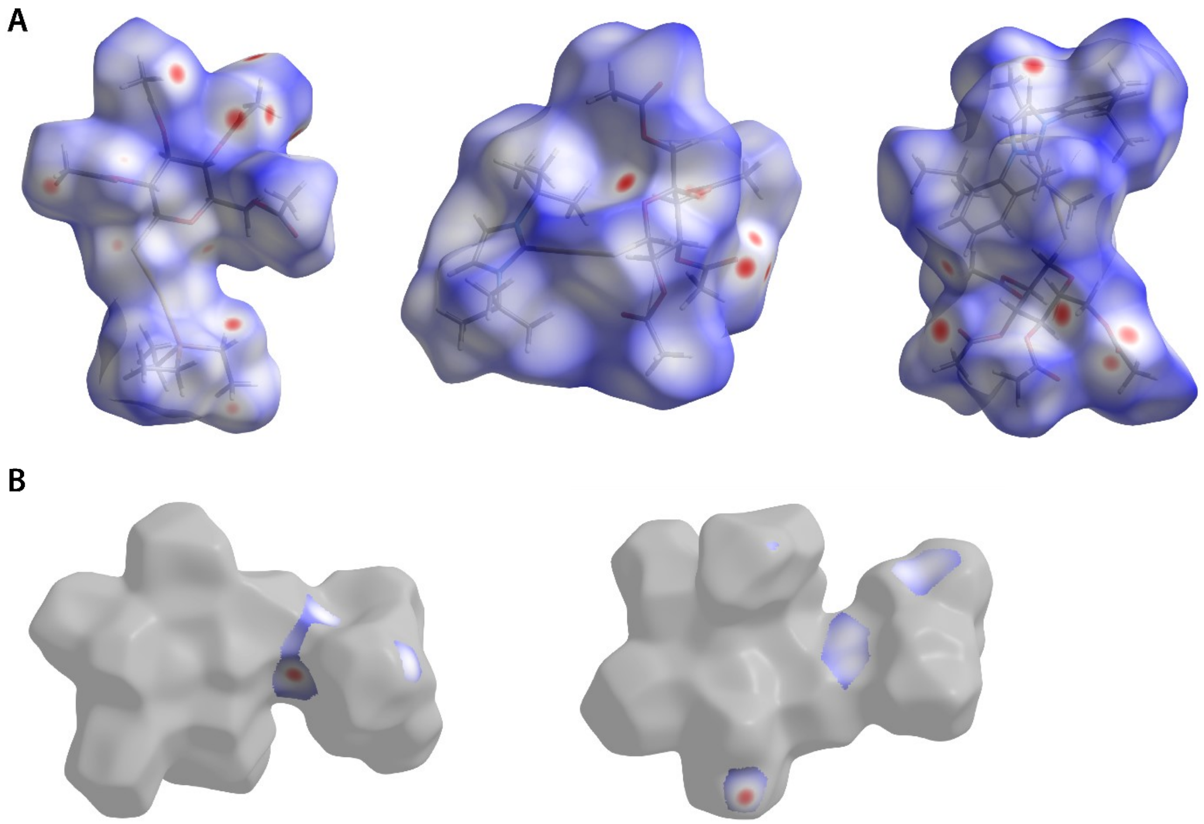


**Fig. S4**. Hirshfeld analysis. **A.** HS mapped onto $d_e$ for **AF** (left), ECIJEG (middle) and VEWVOJ (right) Intermolecular contacts which are closer than the sum of their Van der Waals radii are highlighted in red on the surface, whereas longer contacts are blue. Contacts having similar lengths to the radii sum are depicted as white. **B.** Regions of **AF** HS representing Au···H interatomic contacts.

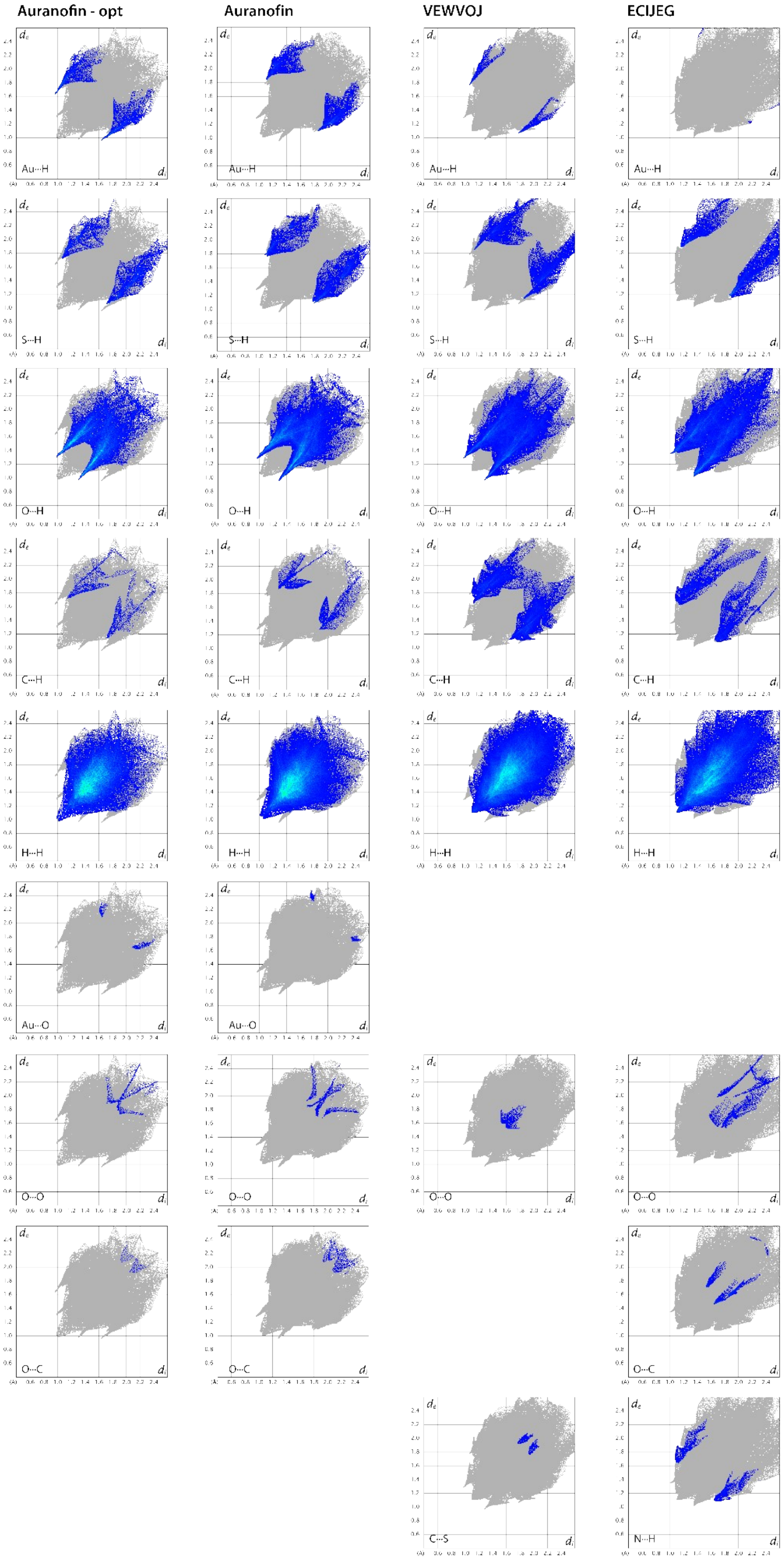


**Fig. S5**. Contribution of different X···X interatomic contacts for selected crystal structures, Auranofin-opt relates to auranofin crystal after geometry optimisation via periodic DFT.

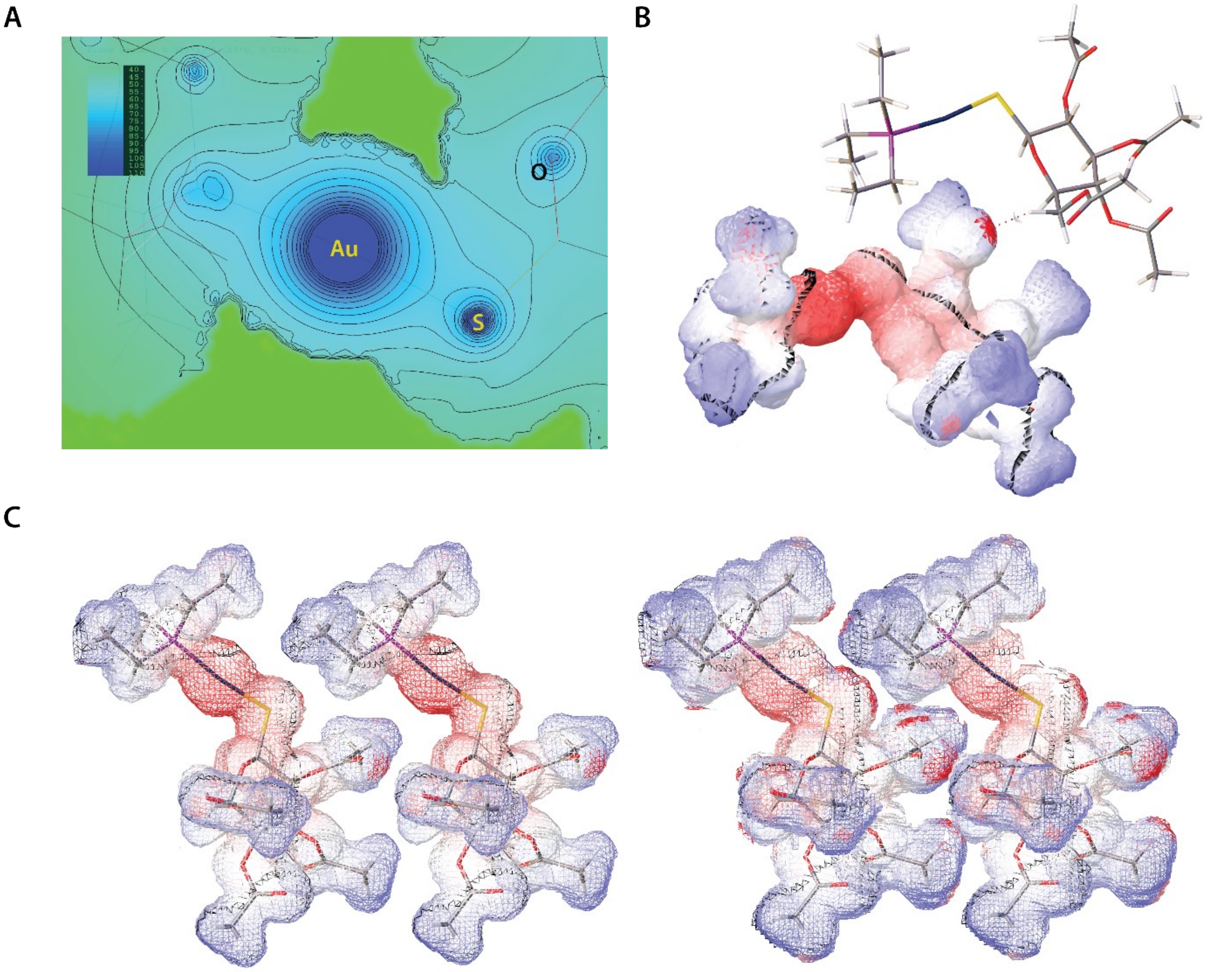


**Fig. S6**. Electrostatic potential for auranofin molecule in the crystal structure. All physical parameters are described in atomic units. **B.** 3D dimensional representation of ESP mapped onto ED isosurface using BWR colouring scheme (contour 0.03 *e* A$^{-3}$) , where low ESP values are blue and high are red (gradient from 10 to 70 $E_h$ $e^{-1}$). Selected intermolecular interactions are depicted as dotted lines. **C.** ESP for a selected dimer of auranofin (the same gradient as in **B** panel) for contour 0.03 *e* A$^{-3}$ (left) and 0.02 *e* A$^{-3}$ (right).

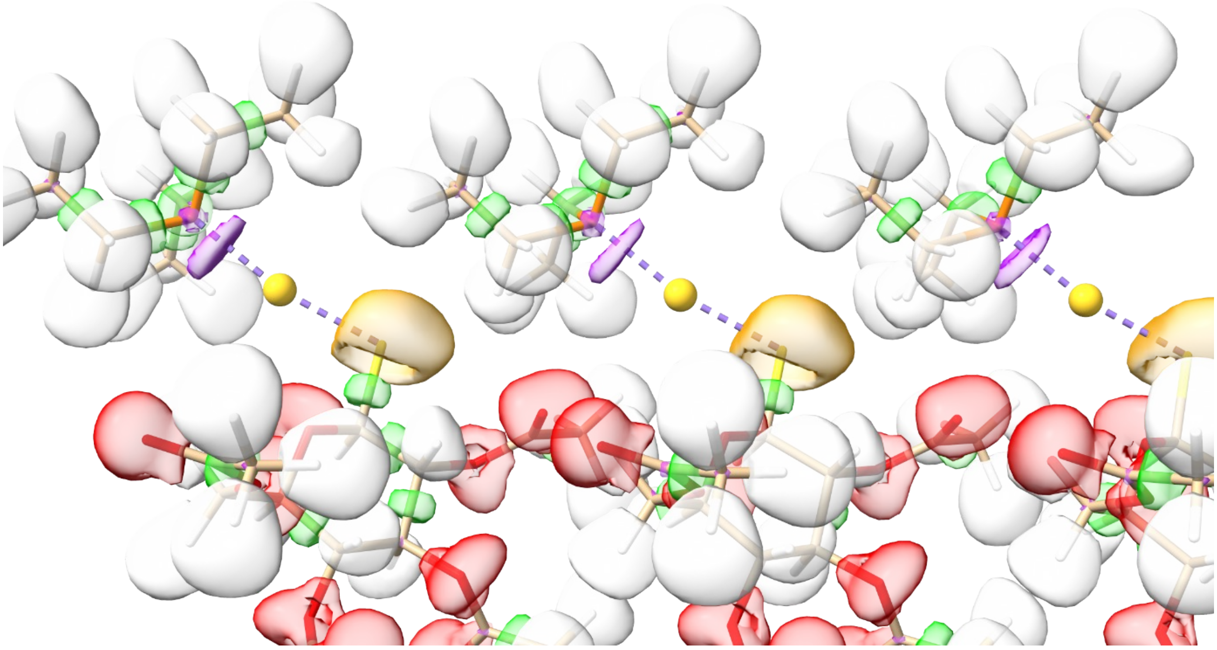

**Fig. S7**. ELF computed for an AF trimer. Different basins are coloured to distinguish different regions of the electronic structure: covalent non-polar bonding (green), lone electron pairs of oxygen (red), deformed electron pairs of sulfur (orange) and hydrogen-related domains (white).

A

B

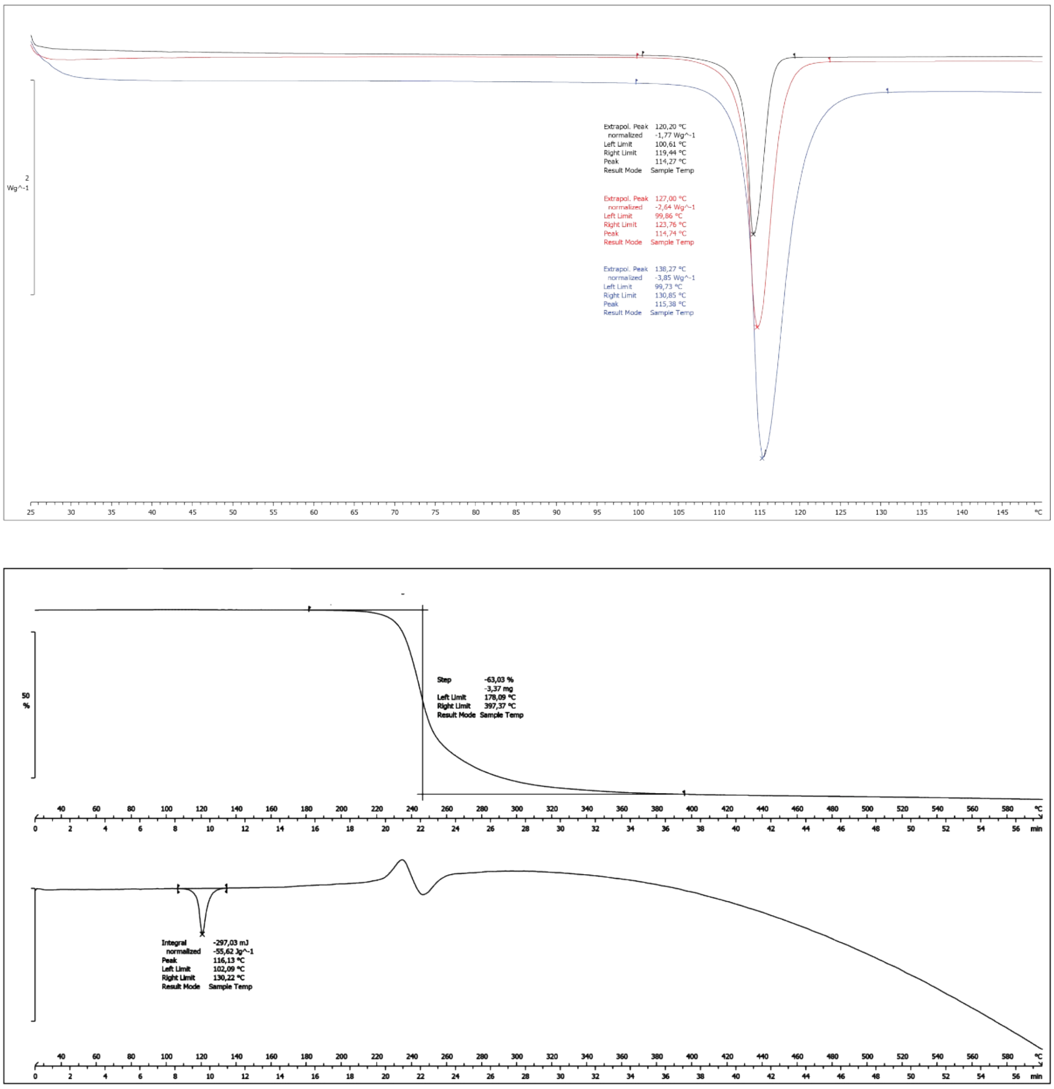


**Fig. S8**. **A.** DSC melting curves of auranofin crystals measured for heat rating of 5, 10 and 20 K min$^{-1}$ (black, red and blue colours respectively). **B.** TGA-DSC profile of auranofin crystals.

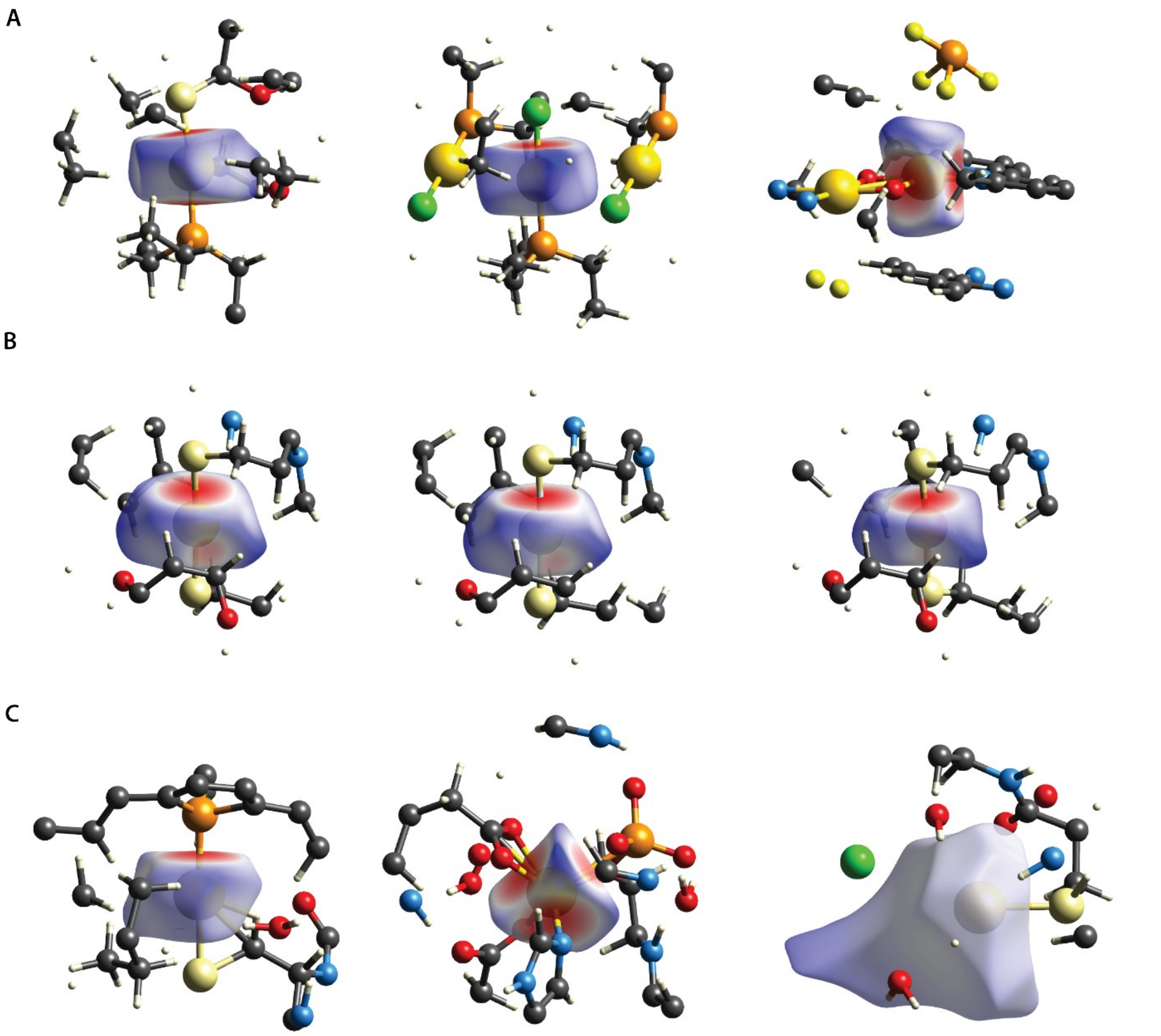


**Fig. S9**. Comparison between HS of Au(I) atoms and monovalent cations in complex compounds and metal-protein complexes. **A**. Au atoms in complex compounds AF (left), triphenylphosphine, SATTEM (centre) and QIYZII (right). **B**. Metal cations complexed by CueR. $Au^+$ (left), $Ag^+$ (centre), and $Cu^+$ (right). **C**. $Au^+$ complexed by human glutathione reductase (left), bacterial enzyme MRC-1-S (centre) and *E. Histolytica* thioredoxin reductase (right).

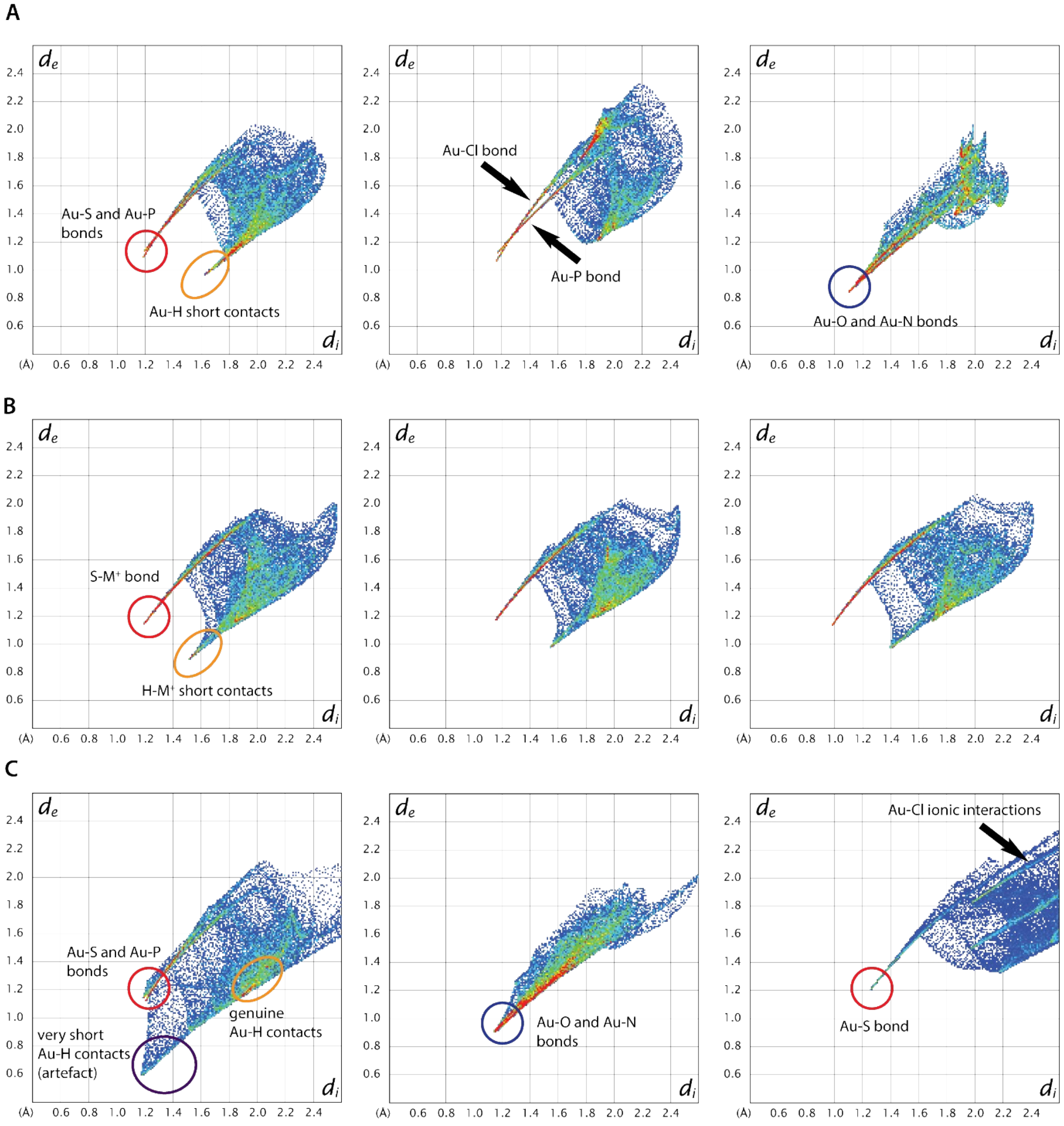


**Fig. S10**. Comparison between fingerprint plots of Au atoms or cations in complex compounds and metal-protein complexes. **A**. Au atoms in complex compounds AF (left), triphenylphosphine, SATTEM (centre) and QIYZII (right). **B**. Metal cations complexed by CueR. $Au^+$ (left), $Ag^+$ (centre), and $Cu^+$ (right). **C**. $Au^+$ complexed by human glutathione reductase (left), bacterial enzyme MRC-1-S (centre) and *E. Histolytica* thioredoxin reductase (right).
